# Differences in clinical features between axial psoriatic arthritis and axial spondyloarthritis: a systematic review and meta-analysis of observational studies

**DOI:** 10.3389/fmed.2026.1856665

**Published:** 2026-06-22

**Authors:** Zhihao Qiu, Zijie Chen, Zhe Yang, Ruibo Xia, Weikai Chen, Yuwei Zhu, Weijie Wang, Zhengfu Li, Kepeng Yang

**Affiliations:** 1 The Second School of Clinical Medicine, Zhejiang Chinese Medical University, Hangzhou, China; 2 The First School of Clinical Medicine, Zhejiang Chinese Medical University, Hangzhou, China; 3 Department of Nephrology and Rheumatology, Zhejiang Chinese Medical University, Pingyang Hospital of Traditional Chinese Medicine (Pingyang County Hospital of Traditional Chinese Medicine), Pingyang, China; 4 Rheumatology and Immunology Department, The Second Affiliated Hospital of Zhejiang Chinese Medical University (Xinhua Hospital of Zhejiang Province), Hangzhou, China

**Keywords:** axial psoriatic arthritis, axial spondyloarthritis, bone marrow edema, HLA-B27 testing, spondyloarthritis

## Abstract

**Introduction:**

Classification of axial psoriatic arthritis (ax-PsA) within existing spondyloarthritis (SpA) criteria remains unclear. To clarify this issue, we compared the clinical features of ax-PsA and axial SpA (ax-SpA).

**Methods:**

In this systematic review and meta-analysis, we searched six databases (PubMed, Web of Science, Cochrane Library, Ovid, Scopus, and Embase) and one trial registry (ClinicalTrials.gov) for studies published from inception to April 20, 2025, without language restrictions. Observational studies comparing ax-PsA and ax-SpA were included. Meta-analyses were conducted to evaluate disease activity scores, spinal function scores, inflammatory markers, and HLA-B27 positivity.

**Results:**

Fifteen studies including 2,704 patients with ax-PsA and 9,248 with ax-SpA from diverse global populations were analyzed. Meta-analysis showed no significant differences between ax-PsA and ax-SpA in the Bath Ankylosing Spondylitis Disease Activity Index, Ankylosing Spondylitis Disease Activity Score, Bath Ankylosing Spondylitis Functional Index, or C-reactive protein levels. However, ax-PsA was associated with significantly lower HLA-B27 positivity compared with ax-SpA (risk ratio, 0.37; 95% confidence interval [CI], 0.32–0.43; *p* < 0.001). In subgroup analyses, ax-PsA demonstrated modestly better BASFI scores than ankylosing spondylitis (mean difference, −0.35; 95% CI, −0.64–−0.05; *p* = 0.022).

**Conclusion:**

The marked difference in HLA-B27 positivity between ax-PsA and ax-SpA supports their distinction as separate disease entities. Similar disease activity and functional outcomes suggest shared downstream inflammatory pathways. The lower HLA-B27 positivity in ax-PsA may reflect alternative mechanisms of interleukin (IL)-23/IL-17 axis activation, with potential therapeutic implications. Clinical differentiation should incorporate HLA-B27 testing and imaging. Future research should focus on disease course variability and subgroup-specific characteristics.

**Systematic review registration:**

https://www.crd.york.ac.uk/PROSPERO/view/CRD420251043651, identifier: CRD420251043651.

## 1 Introduction

Psoriatic arthritis (PsA) is a heterogeneous disease characterized by musculoskeletal involvement in addition to skin and nail manifestations, including arthritis, enthesitis, dactylitis, and axial joint involvement (1). As part of the spondyloarthritis (SpA) spectrum, PsA is typically classified as peripheral SpA according to the Assessment of SpondyloArthritis International Society (ASAS) classification criteria (2). Axial involvement occurs in approximately 25–70% of patients with PsA and presents with clinical features similar to those of axial SpA (ax-SpA), including inflammatory back pain, sacroiliitis, and HLA-B27 positivity (3). This subgroup of patients with PsA and axial involvement is commonly referred to as axial PsA (ax-PsA) (4).

However, the classification of ax-PsA remains uncertain. Currently, no standardized diagnostic criteria exist, and existing SpA classification frameworks do not clearly categorize ax-PsA. Consequently, there is ongoing debate as to whether ax-PsA represents a subtype of ax-SpA or a distinct disease entity (5).

Previous studies have detailed the demographic differences between PsA and ax-SpA: compared with ax-SpA, which predominantly affects young males, PsA generally has an older age of onset, a more balanced gender distribution, and a lower HLA-B27 positivity rate (6). Notably, ax-PsA, as a subtype of PsA, exhibits features more similar to ax-SpA than to the overall PsA population, such as a relatively younger age of onset, more pronounced enthesitis, and milder psoriatic manifestations (7). Moreover, some studies suggest that gender plays a similar role in the pathogenesis of ax-PsA and ax-SpA. In both ax-SpA (8) and ax-PsA (9), disease onset in male patients is closely associated with activation of the IL-23/IL-17 pathway and HLA-B27-mediated immune responses, and male patients show greater sensitivity to biologics targeting downstream inflammatory cytokines. Regarding HLA-B27 positivity rates, the literature reports that overall HLA-B27 positivity is approximately 90% in ax-SpA patients, whereas it is significantly lower in ax-PsA patients, at about 40% (10). However, high-quality studies directly comparing ax-PsA and ax-SpA remain limited, and existing conclusions are inconsistent. These inter-study discrepancies need to be further clarified through systematic evidence.

Recent studies have suggested that ax-PsA and ax-SpA may be distinct conditions. Clinical trials have shown that interleukin (IL)-23 inhibitors—biologic agents that have demonstrated limited efficacy in ax-SpA—can improve axial symptoms in patients with ax-PsA (11). In addition, pathological features differ between the two conditions: ax-PsA is associated with lower HLA-B27 positivity and is characterized by para-ligamentous osteophytes and relatively preserved sacroiliac joint spaces, whereas ax-SpA is marked by diffuse bone marrow edema and fatty corner lesions, strongly associated with HLA-B27 (12).

Despite these observations, further clinical evidence is needed. In trials reporting improvement of axial symptoms in ax-PsA with IL-23 inhibitors, primary outcomes included disease activity scores and magnetic resonance imaging (MRI) findings, such as reductions in axial bone marrow edema. However, commonly used disease activity scores are not specific for axial inflammation and may be influenced by improvements in peripheral disease (13). Additionally, MRI-detected bone marrow edema is frequently observed in the general population (14), and imaging evidence of improvement in the characteristic ligamentous inflammation of ax-PsA remains limited. Furthermore, global epidemiological data indicate substantial regional variation in HLA-B27 positivity among patients with ax-SpA (15), suggesting that both genetic and environmental factors contribute to disease expression. Thus, differences in HLA-B27 positivity alone may not fully explain the pathological distinctions between ax-PsA and ax-SpA, particularly given the lack of a clear definition of ax-PsA.

The results of the Axial Involvement in Psoriatic Arthritis study, conducted by the Group for Research and Assessment of Psoriasis and Psoriatic Arthritis in collaboration with ASAS, are forthcoming (16). In this context, we aimed to provide complementary evidence by synthesizing existing observational studies that compare the clinical characteristics of ax-PsA and ax-SpA.

## 2 Materials and methods

### 2.1 Search strategy

This systematic review and meta-analysis was conducted in accordance with the Preferred Reporting Items for Systematic Reviews and Meta-Analyses guidelines. We searched six databases (PubMed, Web of Science, Cochrane Library, Ovid, Scopus, and Embase) and one registry (ClinicalTrials.gov) for studies published from inception to April 20, 2025. No language restrictions were applied. Detailed search strategies are provided in the Supplementary material.

### 2.2 Eligibility criteria

Eligible studies were observational clinical studies that included both ax-PsA and ax-SpA groups. The diagnosis of ax-PsA had to meet both of the following criteria: (1) the 2006 CASPAR criteria for psoriatic arthritis (17); and (2) the diagnosis of axial involvement had to satisfy at least one of two sets of criteria for ax-SpA: either the imaging arm of the 2009 ASAS classification criteria (18) (one SpA feature plus imaging positivity, with imaging evidence including X-ray or MRI) or the 1984 Modified New York criteria (19) (mNY, i.e., radiographic sacroiliitis). The ax-SpA group had to meet either the 2009 ASAS classification criteria or the 1984 Modified New York criteria. Studies were required to report disease activity scores for both groups. Studies were excluded if the criteria for disease activity scoring were unclear or if relevant data were missing.

### 2.3 Literature screening and data extraction

During literature screening, records with critical missing information were first excluded, followed by removal of duplicates. Titles and abstracts were screened to exclude meta-analyses, systematic reviews, reviews, case reports or case series, experimental studies (*in vivo* or *in vitro*), letters, meeting abstracts, clinical guidelines, expert consensus statements, corrections, editorials, protocols, and studies outside the scope of this review.

The full texts of the remaining articles were retrieved and assessed for eligibility. Inclusion was based on: (1) clarity of diagnostic definitions for ax-PsA and ax-SpA; (2) reporting of disease activity scores for both groups; (3) appropriateness of scoring criteria; and (4) avoidance of duplicate data from the same cohort across multiple publications. Screening was performed independently by two reviewers (Zhihao Qiu and Zijie Chen), with disagreements resolved by a third reviewer (Zhengfu Li). Data extraction was conducted independently by two additional reviewers (Weikai Chen and Yuwei Zhu), with discrepancies resolved by a third reviewer (Kepeng Yang).

### 2.4 Data analysis

The primary objective was to compare clinical features of ax-PsA and ax-SpA across four domains: disease activity (Bath Ankylosing Spondylitis Disease Activity Index [BASDAI] and Ankylosing Spondylitis Disease Activity Score [ASDAS]), functional status (Bath Ankylosing Spondylitis Functional Index [BASFI]), inflammatory markers (C-reactive protein [CRP] and erythrocyte sedimentation rate [ESR]), and HLA-B27 positivity. Outcomes reported in fewer than five studies were excluded to ensure robustness and enable subgroup analyses.

For continuous outcomes, pooled effect sizes were calculated using standardized mean differences or weighted mean differences based on reported means and standard deviations. Odds ratios were used for dichotomous outcomes. Heterogeneity was assessed using the I^2^ statistic and Cochrane Q-test (*p* < 0.1 indicating statistical significance). Fixed- or random-effects models were applied as appropriate.

In cases of substantial heterogeneity, sensitivity analyses were conducted using a leave-one-out approach. For dichotomous outcomes, L'Abbé and radial plots were generated to further assess heterogeneity. To further explore the sources of heterogeneity, we planned to conduct meta-regression analyses, including covariates covering overall study characteristics and differences in population characteristics between the two groups. Given the potential for an insufficient number of included studies and missing data in this study, only one covariate was entered in each meta-regression (i.e., univariable meta-regression). Covariates showing a strong association with a *p*-value < 0.05 were identified through meta-regression. Categorical covariates were further analyzed using subgroup analyses. For continuous covariates, bubble plots and prediction interval plots were constructed to illustrate their moderating effects. Publication bias was assessed using funnel plots, Egger's test, and Begg's test. Meta-analysis, heterogeneity assessment, sensitivity analysis, subgroup analysis, and publication bias analysis were performed using Stata version 14.0 (College Station, TX, USA). Univariable meta-regression, bubble plots, and prediction interval plots were generated using R version 4.5.3.

### 2.5 Risk of bias assessment

Risk of bias was assessed using the Newcastle-Ottawa Scale (NOS) for cohort studies, with modifications tailored to the objectives of this meta-analysis. In the “Selection” domain, item 4 (“Demonstration that outcome of interest was not present at start of study”) was modified to: “Was ax-PsA assessed for ax-SpA disease activity prior to cohort entry?” A score of one star was assigned for “No,” while “Yes” or “Not described” received zero stars.

Comparability” domain, two items were included: (1) whether diagnostic criteria for ax-PsA and ax-SpA were clearly specified (one star for “Yes”), and (2) whether confounding by peripheral SpA manifestations was controlled (one star for “Yes”). These modifications were made to minimize the influence of peripheral symptoms on disease activity scores, the primary outcomes of this study. Risk of bias assessment was conducted independently by two reviewers (Zhe Yang and Ruibo Xia), with disagreements resolved by a third reviewer (Wang). No automated tools were used.

## 3 Results

Following initial screening of titles and abstracts, 51 potentially relevant articles were identified. After full-text review, studies that did not meet the inclusion criteria were excluded. For example, one study from Egypt was excluded because it did not specify the inflammatory markers used to calculate the ASDAS (20). Another retrospective study from South Korea was excluded because it did not report disease activity scores for the ax-PsA group (21). Ultimately, 16 studies were included in the systematic review. Among these, two publications by José Luis Fernández-Sueiro et al. reported data from the same cohort; after merging these data, 15 studies were included in.

During data extraction, only two studies reported ASDAS-ESR scores. Given evidence that ASDAS-ESR is inferior to ASDAS-CRP for assessing disease activity in ax-SpA and that the two measures are not interchangeable (22), only the BASDAI and ASDAS-CRP were included in the final analysis of disease activity.

For inflammatory markers, only four studies reported the ESRs. As prior studies have suggested that CRP is more reliable than ESR for differentiating axial involvement in SpA-related arthritis (23), CRP was selected as the primary inflammatory marker for meta-analysis.

Due to the limited number of included studies and missing data in some cases, we performed univariable meta-regression analyses, including a total of seven covariates. Regarding overall study characteristics: first, because we adopted two sets of criteria for defining axial involvement in the inclusion criteria, the definition of axial involvement in ax-PsA was included as a covariate. Second, the type of control group was included, as some studies explicitly defined ax-SpA as ankylosing spondylitis (AS). Finally, considering that all included studies were observational and thus subject to inherent random error (24), the total sample size of the study was also included as a covariate. Regarding population characteristics, four covariates were included: sex, age, disease duration, and peripheral arthritis involvement. Categorical covariates that showed a significant association with the effect size were used as the basis for subgroup analyses; further details are provided in the Supplementary material.

The 15 included studies comprised 2,704 patients with ax-PsA and 9,248 patients with ax-SpA. Among the included studies, the mean age of patients in the ax-PsA group ranged from 36.9 to 59.5 years, the proportion of males ranged from 40.0% to 71.9%, disease duration ranged from 1 to 18 years, and the proportion of peripheral arthritis involvement ranged from 0% to 100%. In the ax-SpA group, the mean age ranged from 32.0 to 54.7 years, the proportion of males ranged from 53.0% to 86.4%, disease duration ranged from 5 to 22 years, and the proportion of peripheral arthritis involvement ranged from 0% to 88.9%. Baseline characteristics of the study populations are summarized in Table 1.

**Table 1:** Basic information regarding the included studies.

Research	Patient Source	ax-PsA group						ax-SpA group					
		Diagnostic Criteria	Sample size	Sex	Age	DB	PA	Experimental group	Sample size	Sex	Age	DB	PA
Diego Benavent 2021 [S1]	Global Regions	ASAS	367	53.4%	50.0	9.9	86.6%	ax-SpA	2,651	68.5%	42.1	13.1	35.7%
Diego Benavent 2021 [S2]	Spain	RD	65	66.2%	36.9	8.9	78.5%	ax-SpA	287	62.7%	35.7	7.9	41.5%
Adrian Ciurea 2023 [S3]	Switzerland	RD	1,153	51.0%	48.6	9.8	86.7%	ax-SpA	4,489	53.0%	41.8	9.0	45.8%
Ran Cui 2023 [S4]	China	mNY	68	70.6%	46.0	1.0	NA	AS	446	76.5%	32.0	5.0	NA
Joy Feld 2020 [S5]	Canada	mNY	477	63.5%	45.9	NA	NA	AS	766	72.8%	38.5	NA	NA
José Luis Fernández-Sueiro 2010 [S6]	Spain	mNY	46	NA	NA	NA	NA	AS	103	NA	NA	NA	NA
George E Fragoulis 2022 [S7]	Greece	ASAS	79	45.6%	52.1	6.4	91.1%	AS	129	60.5%	48.9	8.1	42.6%
E E Gubar 2025 [S8]	Russia	mNY	55	61.8%	45.5	5.0	100.0%	ax-SpA	45	53.3%	34.9	6.0	88.9%
Muhammad Haroon 2018 [S9]	Ireland	ASAS	15	40.0%	38.6	4.5	NA	AS	15	53.3%	33.9	5.4	NA
Deepak R Jadon 2017 [S10]	United Kingdom	mNY	117	63.3%	59.5	18	NA	AS	157	75.2%	54.7	22	NA
Arthur Kavanaugh 2023 [S11]	Global Regions	RD	190	66.3%	44.6	NA	NA	AS	323	86.4%	40.0	NA	NA
Kwok et al. (25) [S12]	Canada	mNY	32	71.9%	43.1	5.7	0.0%	AS	82	73.7%	36.9	7.3	0.0%
Xabier Michelena 2022 [S13]	Spain	RD	109	62.4%	50.1	7.0	78.0%	AS	127	78.7%	49.9	9.0	58.1%
Gabriel Caetano Pereira 2024 [S14]	Brazil	ASAS	32	68.8%	51.1	15.7	71.9%	ax-SpA	62	59.7%	44.1	15.9	32.3%
Yanushonite A.A. 2024 [S15]	Russia	RD	30	50.0%	49.1	12.6	90.0%	ax-SpA	30	53.3%	42.9	16.3	36.7%

[S1],[S2],…,[S15] refer to the corresponding entries in the Supplementary material, where the full citation details (including DOI) are provided; Diagnostic Criteria refers to the diagnostic criteria for axial involvement in ax-PsA; Sex is the proportion of males; Age is the current mean age; DB is the abbreviation for disease duration, referring to the mean disease duration after the onset of inflammatory back pain; PA stands for peripheral arthritis, referring to the proportion of patients with peripheral arthritis; ASAS refers to the 2009 ASAS classification criteria for ax-SpA; mNY refers to the 1984 Modified New York criteria; RD is the abbreviation for rheumatologist's diagnosis, indicating axial involvement determined by a rheumatologist based on clinical criteria and the patient's ax-PsA status; NA indicates missing data; All values are rounded to one decimal place.

**Figure 1:**
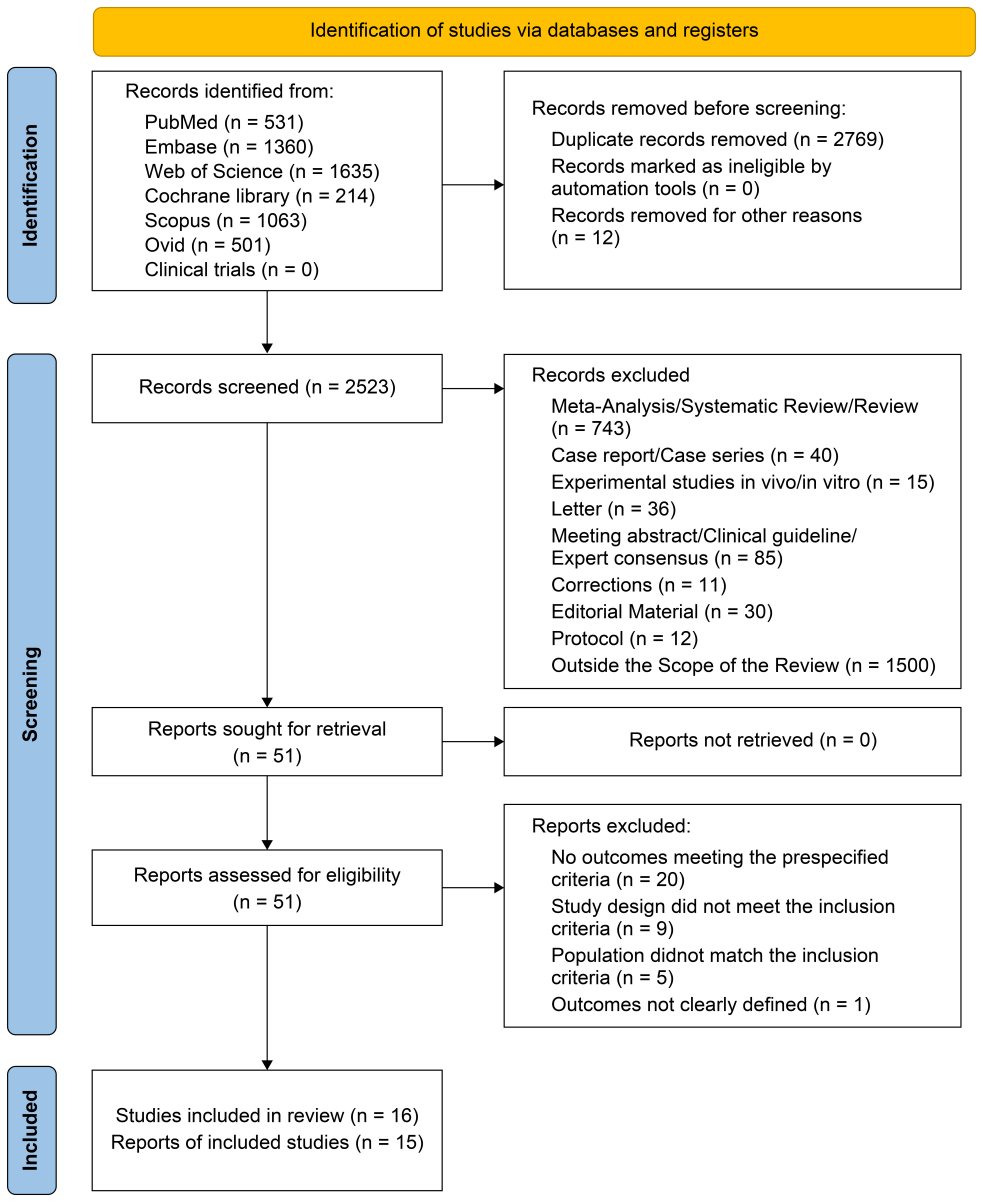
Literature screening process (*Among the 16 studies ultimately included, two were based on the same patient cohort; therefore, only 15 studies were reported in the final analysis.).

The literature selection process is shown in Figure 1. The risk of bias assessment (Table 2) showed that all included studies had a NOS score of ≥ 7, indicating high overall methodological quality.

**Table 2:** Quality assessment based on the Newcastle-Ottawa Scale.

Research	Selection	Comparability	Explore	Total
Diego Benavent 2021 [S1]	⋆⋆⋆⋆	⋆⋆	⋆⋆⋆	9⋆
Diego Benavent 2020 [S2]	⋆⋆⋆	⋆	⋆⋆⋆	7⋆
Adrian Ciurea 2023 [S3]	⋆⋆⋆	⋆⋆	⋆⋆	7⋆
Ran Cui 2023 [S4]	⋆⋆⋆	⋆	⋆⋆⋆	7⋆
Joy Feld 2020 [S5]	⋆⋆⋆	⋆⋆	⋆⋆⋆	8⋆
Jose' Luis Fernández-Sueiro 2010 [S6]	⋆⋆⋆	⋆	⋆⋆⋆	7⋆
G.E. Fragoulis 2022 [S7]	⋆⋆⋆	⋆	⋆⋆⋆	7⋆
E. E. Gubar 2025 [S8]	⋆⋆⋆⋆	⋆	⋆⋆⋆	8⋆
Muhammad Haroon 2018 [S9]	⋆⋆⋆⋆	⋆	⋆⋆⋆	8⋆
Deepak R Jadon 2017 [S10]	⋆⋆⋆⋆	⋆	⋆⋆⋆	8⋆
Arthur Kavanaugh 2023 [S11]	⋆⋆⋆⋆	⋆	⋆⋆⋆	8⋆
Kwok et al. (25) [S12]	⋆⋆⋆	⋆	⋆⋆⋆	7⋆
Xabier Michelena 2022 [S13]	⋆⋆⋆⋆	⋆	⋆⋆⋆	8⋆
Gabriel Caetano Pereira 2024 [S14]	⋆⋆⋆⋆	⋆	⋆⋆⋆	8⋆
Yanushonite A.A. 2024 [S15]	⋆⋆⋆⋆	⋆	⋆⋆⋆	8⋆

⋆ indicates that one point was awarded for meeting the corresponding criterion. The total score is the sum of stars achieved, ranging from 0 to 8 (one point per item). Studies with higher scores are considered to have lower risk of bias.

### 3.1 Disease activity

**Figure 2:**
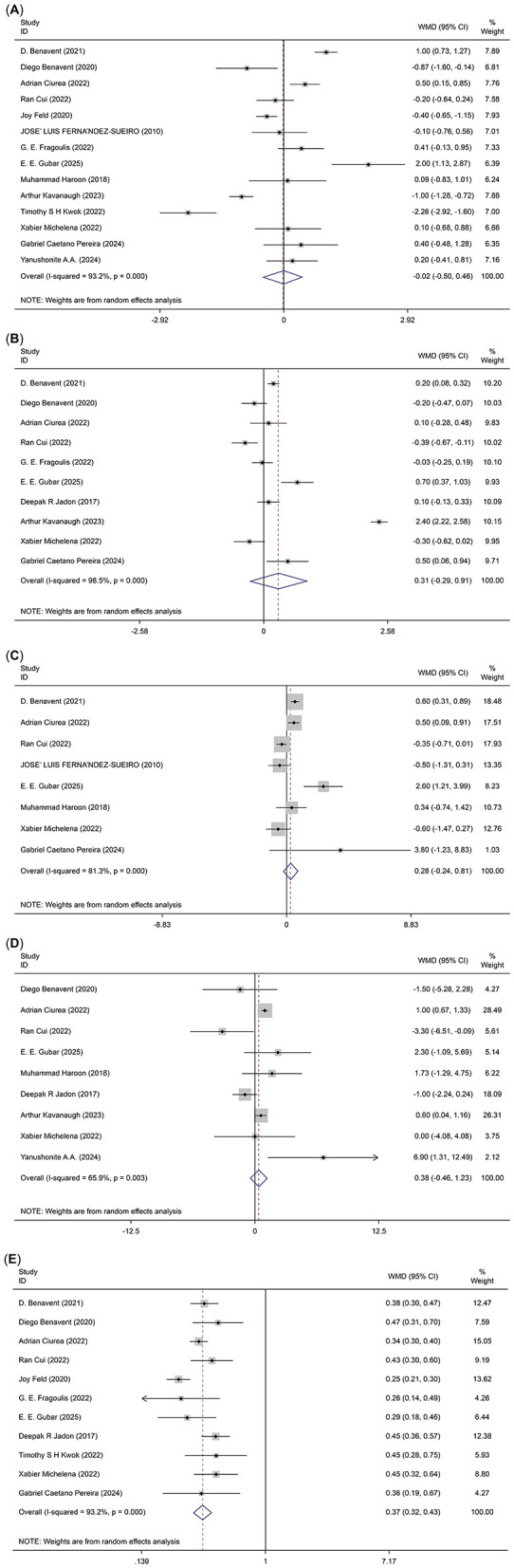
Results of the meta-analyses: **(A)** BASDAI score, **(B)** ASDAS-CRP score, **(C)** BASFI score, **(D)** CRP level, and **(E)** Relative risk (RR) for HLA-B27 positivity.

**Figure 3:**
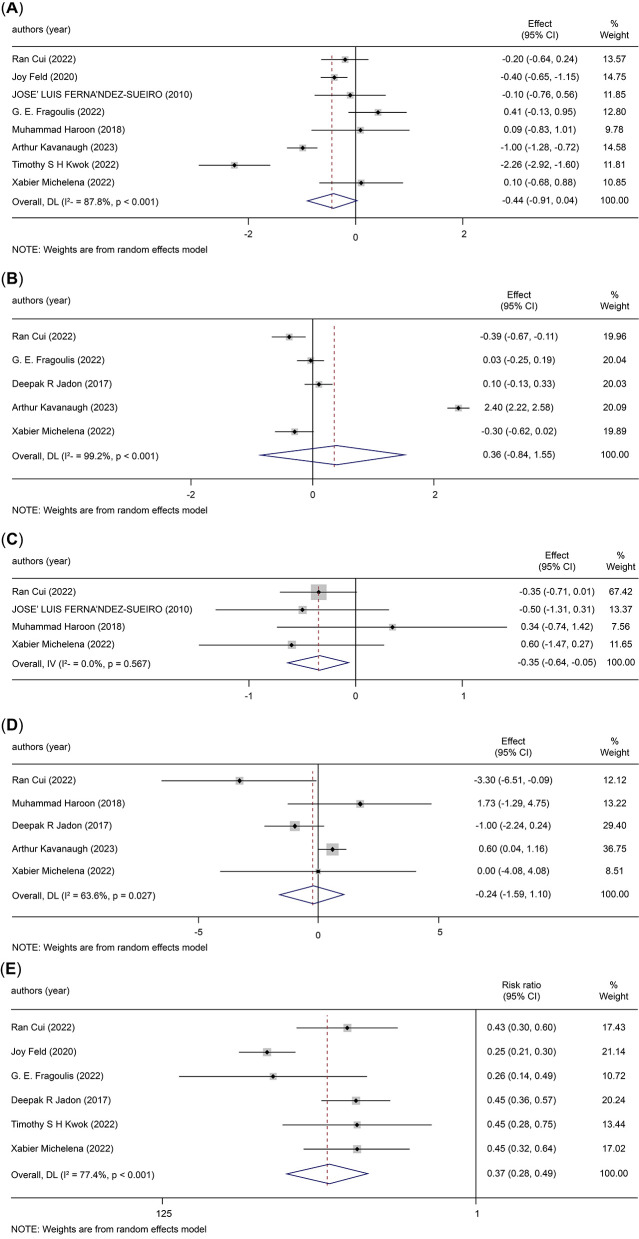
Subgroup analysis restricted to ankylosing spondylitis based on the **(A)** BASDAI score, **(B)** ASDAS-CRP score, **(C)** BASFI score, **(D)** CRP level, and **(E)** Relative risk (RR) for HLA-B27 positivity.

**Figure 4:**
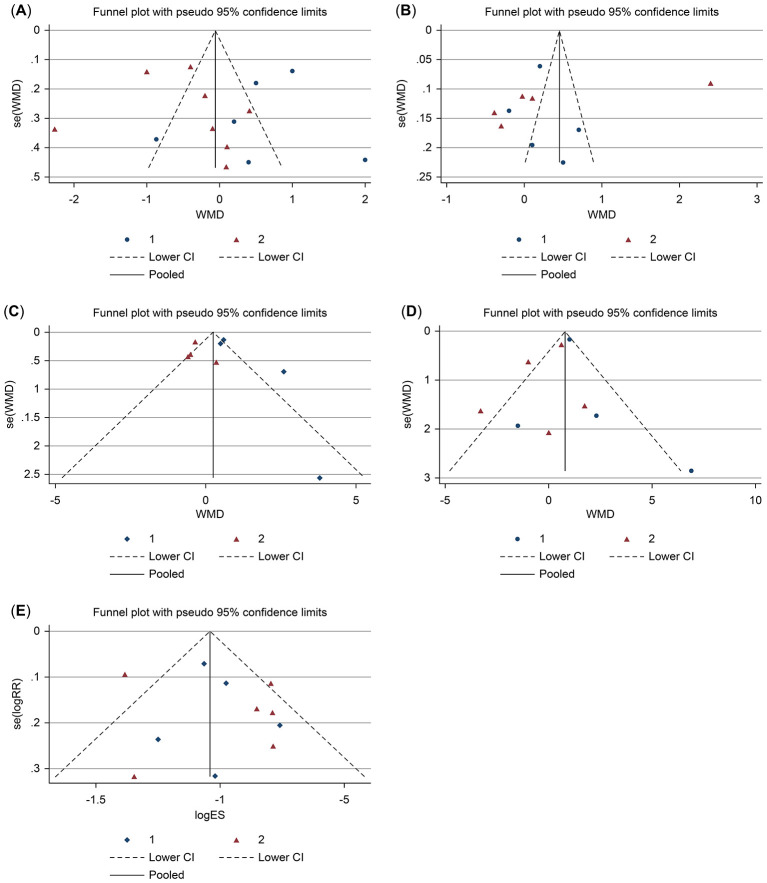
Funnel plots for publication bias assessment: **(A)** BASDAI score, **(B)** ASDAS-CRP score, **(C)** BASFI score, **(D)** CRP level, and **(E)** Relative risk (RR) for HLA-B27 positivity.

In this meta-analysis, 14 studies reported BASDAI scores. Heterogeneity testing revealed an I^2^ value of 93.2% and a Q-test *p*-value < 0.001, indicating substantial heterogeneity among the included studies. Sensitivity analysis did not identify any single study as a clear source of heterogeneity; therefore, a random-effects model was used to pool the results. The pooled effect size was −0.02 (−0.50–0.46), which was not statistically significant (Z = 0.09, *p* = 0.926). There was no significant difference in BASDAI scores between the ax-PsA and ax-SpA groups (Figure 2A). Furthermore, meta-regression analysis did not identify a source of heterogeneity. Based on clinical relevance, we performed a subgroup analysis restricted to studies in which the control group comprised patients with AS. The results (Figure 3A) continued to show high heterogeneity within the AS subgroup (I^2^ = 87.8%, Q-test *p* < 0.001). The pooled effect size using a random-effects model was −0.439 (−0.914–0.037), which was not statistically significant (Z = 1.81, *p* = 0.070). Thus, no significant difference in BASDAI scores was observed between ax-PsA and either the broader ax-SpA group or the AS subgroup. Publication bias assessment using a funnel plot (Figure 4A), along with Egger's test (*p* = 0.886) and Begg's test (*p* = 0.784), indicated symmetry, supporting the reliability of the findings.

Regarding ASDAS-CRP scores, 10 studies were included. Heterogeneity testing showed an I^2^ of 98.5% with a Q-test *p*-value < 0.001, indicating very high heterogeneity. Sensitivity analysis did not identify a specific study as the source. Using a random-effects model, the pooled effect size was 0.31 (−0.29–0.91), which was not statistically significant (Z = 1.00, *p* = 0.315). Therefore, no significant difference in ASDAS-CRP scores was observed between the ax-PsA and ax-SpA groups (Figure 2B). Meta-regression analysis again did not identify a clear source of heterogeneity. In the subgroup analysis restricted to AS, heterogeneity remained extremely high (I^2^ = 99.2%, Q-test *p* < 0.001). The pooled effect size using a random-effects model was 0.359 (−0.294–0.914), which was not statistically significant (Z = 0.59, *p* = 0.556) (Figure 3B). Thus, no significant differences in ASDAS-CRP scores were found when comparing ax-PsA with either the overall ax-SpA group or the AS subgroup. Assessment of publication bias using a funnel plot (Figure 4B), together with Egger's test (*p* = 0.612) and Begg's test (*p* = 0.421), indicated symmetry, further supporting the robustness of these findings.

### 3.2 Physical function

BASFI scores were reported in eight studies. Heterogeneity testing showed I^2^ = 81.3% with a Q-test *p*-value < 0.001, indicating moderate to substantial and statistically significant heterogeneity. Sensitivity analysis did not identify any influential study. Using a random-effects model, the pooled effect size was 0.28 (−0.24–0.81), which was not statistically significant (Z = 1.05, *p* = 0.292). This indicates no significant difference in BASFI scores between the ax-PsA and ax-SpA groups (Figure 2C). Meta-regression analysis suggested that the type of control group may be a source of heterogeneity (*p* = 0.011). Accordingly, subgroup analysis was performed based on control group type. In the AS subgroup, heterogeneity was low (I^2^ = 0%, Q-test *p* = 0.567). Using a fixed-effects model, the pooled mean difference was −0.35 (−0.64–−0.05), which was statistically significant (Z = 2.3, *p* = 0.022) (Figure 3C). This indicates that patients with ax-PsA had significantly lower BASFI scores (i.e., better physical function) than those in the AS group. The funnel plot (Figure 4C) and statistical tests (Egger's test, *p* = 0.158; Begg's test, *p* = 0.497) showed no evidence of publication bias.

### 3.3 Inflammatory markers

Nine studies reported CRP values. Heterogeneity testing showed I^2^ = 65.9% with a Q-test *p*-value of 0.003, indicating moderate and statistically significant heterogeneity. No single study was identified as the source. Using a random-effects model, the pooled effect size was 0.38 (−0.46–1.23), which was not statistically significant (Z = 0.89, *p* = 0.372) (Figure 2D). Meta-regression results were not significant. Subgroup analysis restricted to the AS group showed persistent heterogeneity (I^2^ = 63.6%, Q-test *p* = 0.027). The pooled effect size using a random-effects model was −0.245 (−1.591–1.101), which was not statistically significant (Z = 0.36, *p* = 0.722) (Figure 3D). Thus, no significant differences in CRP levels were observed between ax-PsA and either ax-SpA or AS groups. The funnel plot (Figure 4D) and statistical tests (Egger's *p* = 0.525; Begg's *p* = 0.677) indicated symmetry, supporting the reliability of these findings.

### 3.4 HLA-B27

Eleven studies reported HLA-B27 positivity rates. Heterogeneity testing showed I^2^ = 61% with a Q-test *p*-value < 0.1, indicating significant heterogeneity. L'Abbé plots, radial plots, and sensitivity analyses did not identify any single study as the primary source. Using a random-effects model, the pooled risk ratio (RR) was 0.37 (0.32–0.43), which was statistically significant (Z = 13.29, *p* < 0.001) (Figure 2E). Meta-regression did not identify a suitable variable for further subgrouping. Subgroup analysis restricted to the AS group yielded a pooled RR of 0.37 (0.28–0.50), which remained statistically significant (Z = 6.79, *p* < 0.001) (Figure 3E). The funnel plot (Figure 4E) was symmetrical (Egger's test, *p* = 0.448), indicating no significant publication bias and supporting the robustness of the findings.

## 4 Discussion

The baseline characteristics of the study population showed that the mean age range of patients with ax-PsA was generally higher than that of patients with ax-SpA, while the range of male proportion was slightly lower. Regarding disease duration, ax-PsA patients had a shorter overall disease duration, which may be related to the relatively later occurrence or delayed diagnosis of axial involvement after the onset of psoriasis. In terms of peripheral arthritis, the range of peripheral arthritis proportion in the ax-PsA group showed greater variability than that in the ax-SpA group, reflecting the diversity of clinical phenotypes in ax-PsA.

This meta-analysis is the first to report that the HLA-B27 positivity rate in ax-PsA is only 37% of that in ax-SpA. This finding aligns with existing epidemiological perspectives and further supports the notion that ax-PsA and ax-SpA are distinct clinical entities. Previous studies have indicated that misfolding of the HLA-B27 protein can trigger endoplasmic reticulum stress, leading peripheral macrophages to secrete IL-23 and activate downstream inflammatory pathways (26). The lower HLA-B27 positivity observed in the ax-PsA population in this study suggests that the IL-23/IL-17 inflammatory signaling axis in this group may be activated via pathways independent of HLA-B27 expression. This provides a mechanistic explanation for the alleviation of axial joint inflammation through inhibition of IL-23 and its downstream inflammatory mediators. HLA-B27 is a key discriminant between ax-PsA and ax-SpA and may be involved in directing targeted damage in the axial joints of ax-SpA, thereby partially explaining differences in their pathological manifestations.

Notably, apart from HLA-B27 positivity, no significant differences were found between ax-PsA and ax-SpA in BASDAI, ASDAS-CRP, BASFI scores, or CRP levels. Previous studies have shown that ASDAS (27) and CRP (28) reflect the extent of active spinal inflammation in ax-SpA as assessed by MRI. The BASDAI score is associated with neck pain, whereas the BASFI score is closely related to stiffness (29). Our results indicate that ax-PsA and ax-SpA exhibit similar severity in terms of spinal inflammation, neck pain, and low back stiffness, which may be related to shared downstream inflammatory pathways. Thus, clinical differentiation between the two conditions still relies on HLA-B27 status and imaging findings.

Although the overall comparison showed no significant difference in BASFI scores between ax-PsA and ax-SpA, meta-regression identified control group type as a source of heterogeneity. Further subgroup analysis using AS as the control group revealed that patients with ax-PsA had lower BASFI scores than those in the AS group, with a mean difference of −0.35 (−0.64–−0.05). Notably, a previous meta-analysis reported no significant difference in BASFI scores between non-radiographic ax-SpA (nr-ax-SpA) and AS (30), suggesting that the lack of an overall difference in our study was not due to confounding from the nr-ax-SpA population. Epidemiological surveys have shown that patients with ax-PsA are predominantly younger male individuals with shorter disease duration and less extensive axial involvement (31). Therefore, we speculate that disease duration may be an important factor influencing functional scores. Previous studies have reported that, in ax-SpA, shorter disease duration is associated with radiographic changes and spinal function (32), and that inflammatory markers such as CRP or ESR, when combined with disease duration, better reflect limitations in axial joint function (33). Thus, disease duration is a significant confounder in assessing axial function in patients with ax-SpA and should be carefully considered in future observational studies comparing ax-PsA and ax-SpA.

However, further meta-regression with disease duration as a continuous covariate did not reveal a significant association. To further explore the relationship between disease duration and the effect size, we constructed bubble plots and prediction interval plots (see Supplementary material for details). The bubble plots showed that data points from individual studies were scattered across the range of disease duration differences, and the 95% confidence band of the regression line included the zero-effect line across most of the range. Similarly, the prediction interval plots did not show a clear increasing or decreasing trend in the predicted effect size as the disease duration difference varied, and the prediction intervals were wide. These visual results further support the absence of a significant linear association between disease duration and the BASFI effect size. Several explanations may account for this apparent discrepancy. First, disease duration data were missing for many studies (only 9 out of 15 studies reported usable disease duration data, a missing rate of approximately 40%), which reduced the statistical power of the meta-regression and the graphical analyses. Second, differences in the definition of disease duration across studies may have attenuated the true effect of disease duration. Third, there may be collinearity between disease duration and control group type, as patients in the AS group tended to have longer disease duration, making control group type a stronger predictor in the meta-regression. Fourth, the limited number of included studies (*n* = 15) meant that univariable regression itself had low power to detect even a moderate-strength association. Fifth, the relationship between disease duration and BASFI may not be linear, whereas our study assumed a linear relationship; the bubble plots also suggested a possible non-linear trend, with fluctuations in the data points in the middle range of disease duration. Therefore, although the meta-regression and visual analyses did not confirm a linear association, disease duration should still be considered an important confounder based on previous literature and clinical reasoning, and caution is needed when interpreting the BASFI results.

This study has several limitations. First, owing to inconsistent reporting of outcome measures across the included studies, this meta-analysis was limited to comparisons based on disease activity scores, spinal function scores, systemic inflammatory markers, and HLA-B27 positivity. The ASAS core outcome set for ax-SpA lacks measures of pain and overall health (34), which is a limitation. Second, substantial heterogeneity was observed across multiple outcomes. Although we explored potential sources through sensitivity analyses, meta-regression, and subgroup analyses, residual heterogeneity suggests the influence of other unmeasured factors. Third, we attempted to use disease duration to explain why ax-PsA patients had lower BASFI scores than those in the AS group, but the association between disease duration and BASFI score was weak in the meta-regression. Although we subsequently analyzed possible reasons in detail, high-quality studies are still needed to verify this finding. Finally, regarding study design, only a subset of included studies reported subgroups of patients with ax-SpA or AS with comorbid psoriasis. Such subgroups would be valuable for investigating the temporal relationship between psoriasis onset and axial symptoms. Furthermore, a very small proportion of patients with ax-PsA present with isolated axial symptoms, and their clinical features may closely resemble those of ax-SpA (35). However, among the included studies, only one reported on this subgroup (25), limiting more detailed analysis. Further investigations are needed to address these gaps in future clinical studies.

In conclusion, this meta-analysis confirmed that the HLA-B27 positivity rate is significantly lower in ax-PsA than in ax-SpA, providing further support for their classification as distinct disease entities. Although the two conditions demonstrated similar levels of disease activity, spinal inflammation, and functional scores—suggesting a shared terminal inflammatory pathway—the marked difference in HLA-B27 positivity implies that the IL-23/IL-17 inflammatory axis in ax-PsA may be activated via alternative mechanisms. This finding provides a theoretical basis for therapies targeting this pathway.

These results highlight the importance of combining HLA-B27 testing with imaging for clinical differentiation. Furthermore, disease duration may be a key confounding factor influencing spinal function scores and should be accounted for in future studies. Future research should investigate the temporal relationship between the onset of psoriasis and axial symptoms and further characterize the subgroup of ax-PsA patients presenting with isolated axial involvement.

## Data Availability

The original contributions presented in the study are included in the article/Supplementary material, further inquiries can be directed to the corresponding authors.
