# Supplementary material for: Differences in clinical features between axial psoriatic arthritis and axial spondyloarthritis: a systematic review and meta-analysis of observational studies
Source: Front Med (Lausanne). 2026 Jun 22;13:1856665. doi: 10.3389/fmed.2026.1856665 (PMC13333385; doi:10.3389/fmed.2026.1856665)
Supplement: Supplementary file 1 [file Data_Sheet_1.docx]

***Supplementary Material***

1. **Search**
   1. **Date Searched: April 20, 2025**
   2. **Search Strategy**

Pubmed: ((("Arthritis, Psoriatic"[Mesh]) OR ((((((((Psoriasis Arthropathica[Title/Abstract]) OR (Psoriasis, Arthritic[Title/Abstract])) OR (Arthritic Psoriasis[Title/Abstract])) OR (Psoriatic Arthropathy[Title/Abstract])) OR (Arthropathies, Psoriatic[Title/Abstract])) OR (Arthropathy, Psoriatic[Title/Abstract])) OR (Psoriatic Arthropathies[Title/Abstract])) OR (Psoriatic Arthritis[Title/Abstract]))) AND (axial[Title/Abstract])) AND (("Axial Spondyloarthritis"[Mesh]) OR ((((((Axial Spondyloarthritides[Title/Abstract]) OR (Spondyloarthritides, Axial[Title/Abstract])) OR (Spondyloarthritis, Axial[Title/Abstract])) OR (AxSpA[Title/Abstract])) OR (Non-Radiographic Axial Spondyloarthritis[Title/Abstract])) OR (Spondylitis, Ankylosing[Title/Abstract])))

Web of science:

#5 #4 and #3

#4 TS=(Axial Spondyloarthritis or Axial Spondyloarthritides or Spondyloarthritides, Axial or Spondyloarthritis, Axial or AxSpA or Non-Radiographic Axial Spondyloarthritis or Spondylitis, Ankylosing)

#3 #1 and #2

#2 TS=(axial)

#1 TS=(Arthritis, Psoriatic OR Psoriasis Arthropathica OR Psoriasis, Arthritic OR Arthritic Psoriasis OR Psoriatic Arthropathy OR Arthropathies, Psoriatic OR Arthropathy, Psoriatic OR Psoriatic Arthropathies OR Psoriatic Arthritis)

Ovid: ((Arthritis, Psoriatic or Psoriasis Arthropathica or Psoriasis, Arthritic or Arthritic Psoriasis or Psoriatic Arthropathy or Arthropathies, Psoriatic or Arthropathy, Psoriatic or Psoriatic Arthropathies or Psoriatic Arthritis).ab,ti AND (axial).ab,ti) AND (Axial Spondyloarthritis or Axial Spondyloarthritides or Spondyloarthritides, Axial or Spondyloarthritis, Axial or AxSpA or Non-Radiographic Axial Spondyloarthritis or Spondylitis, Ankylosing).ab,ti

Scopus: ( TITLE-ABS-KEY ( "Arthritis, Psoriatic" OR "Psoriasis Arthropathica" OR "Psoriasis, Arthritic" OR "Arthritic Psoriasis" OR "Psoriatic Arthropathy" OR "Arthropathies, Psoriatic" OR "Arthropathy, Psoriatic" OR "Psoriatic Arthropathies" OR "Psoriatic Arthritis" ) AND TITLE-ABS-KEY ( "axial" ) ) AND TITLE-ABS-KEY ( "Axial Spondyloarthritis" OR "Axial Spondyloarthritides" OR "Spondyloarthritides, Axial" OR "Spondyloarthritis, Axial" OR "AxSpA" OR "Non-Radiographic Axial Spondyloarthritis" OR "Spondylitis, Ankylosing" )

Embase:

#9 #5 AND #8

#8 #6 OR #7

#7 'axial spondyloarthritides':ab,ti OR 'spondyloarthritides, axial':ab,ti OR 'spondyloarthritis, axial':ab,ti OR 'axspa':ab,ti OR 'non-radiographic axial spondyloarthritis':ab,ti OR 'spondylitis, ankylosing':ab,ti

#6 'axial spondyloarthritis'

#5 #3 AND #4

#4 'axial':ab,ti

#3 #1 OR #2

#2 'psoriasis arthropathica':ab,ti OR 'psoriasis, arthritic':ab,ti OR 'arthritic psoriasis':ab,ti OR 'psoriatic arthropathy':ab,ti OR 'arthropathies, psoriatic':ab,ti OR 'arthropathy, psoriatic':ab,ti OR 'psoriatic arthropathies':ab,ti OR 'psoriatic arthritis':ab,ti

#1 ('arthritis,'/exp OR arthritis,) AND psoriatic

Cochrane library:

#1 Arthritis, Psoriatic

#2 (Psoriasis Arthropathica):ab,ti,kw OR (Psoriasis, Arthritic):ab,ti,kw OR (Arthritic Psoriasis):ab,ti,kw OR (Psoriatic Arthropathy):ab,ti,kw OR (Arthropathies, Psoriatic):ab,ti,kw OR (Arthropathy, Psoriatic):ab,ti,kw OR (Psoriatic Arthropathies):ab,ti,kw OR (Psoriatic Arthritis):ab,ti,kw

#3 #1 OR #2

#4 (axial):ab,ti,kw

#5 #3 and #4

#6 Axial Spondyloarthritis

#7 (Axial Spondyloarthritides):ab,ti,kw OR (Spondyloarthritides, Axial):ab,ti,kw OR (Spondyloarthritis, Axial):ab,ti,kw OR (AxSpA):ab,ti,kw OR (Non-Radiographic Axial Spondyloarthritis):ab,ti,kw OR (Spondylitis, Ankylosing):ab,ti,kw

#8 #6 OR #7

#9 #5 AND #8

Clinicaltrials:

Condition/disease: Axial Psoriatic Arthritis \(axPsA\);

Other terms: Axial Spondyloarthritis \(AxSpA\);

Age: Adult (18 - 64);

Study Type: Observational

Study Results: With results

1. **Reference List of Included Studies**
   1. **Studies Included in the Meta-analysis**

S1 Benavent D, Plasencia C, Poddubnyy D,  Kishimoto M, Proft F, Sawada H, et al. Unveiling axial involvement in psoriatic arthritis: An ancillary analysis of the ASAS-perSpA study. Semin Arthritis Rheum (2021) 51:766–74. [doi: 10.1016/j.semarthrit.2021.04.018](http://10.1016/j.semarthrit.2021.04.018)

S2 Benavent D, Plasencia-Rodriguez C, Franco-Gomez K,  Nieto R, Monjo-Henry I, Peiteado D, et al. Axial spondyloarthritis and axial psoriatic arthritis: similar or different disease spectrum? Ther Adv Musculoskelet Dis (2020) 12: 1759720X20971889. [doi: 10.1177/1759720X20971889](http://10.1177/1759720X20971889)

S3 Ciurea A, Gotschi A, Kissling S, Bernatschek A, Bürki K, Exer P, et al. Characterisation of patients with axial psoriatic arthritis and patients with axial spondyloarthritis and concomitant psoriasis in the SCQM registry. RMD open (2023) 9. [doi: 10.1136/rmdopen-2022-002956](http://10.1136/rmdopen-2022-002956)

S4 Cui R, Wang YL, Tao YL, Tong Q, Chen Z, Dai SM. Platelet to albumin ratio is an independent indicator for disease activity in ankylosing spondylitis. Clin Rheumatol (2023) 42:407–13. [doi: 10.1007/s10067-022-06439-x](http://10.1007/s10067-022-06439-x)

S5 Feld J, Ye JY, Chandran V, Inman RD, Haroon N, Cook R, et al. Is axial psoriatic arthritis distinct from ankylosing spondylitis with and without concomitant psoriasis? Rheumatology (Oxford, England) (2020) 59:1340–6. [doi: 10.1093/rheumatology/kez457](http://10.1093/rheumatology/kez457)

S6 Fernandez-Sueiro JL, Willisch A, Pertega-Diaz S, Tasende JA, Fernández‐López JC, Villar NO, Galdo F, et al. Validity of the bath ankylosing spondylitis disease activity index for the evaluation of disease activity in axial psoriatic arthritis. Arthritis Care Res (Hoboken) (2010) 62:78–85. [doi: 10.1002/acr.20017](http://10.1002/acr.20017)

S7 Fragoulis GE, Pappa M, Evangelatos G, Iliopoulos A, Sfikakis PP, Tektonidou MG. Axial psoriatic arthritis and ankylosing spondylitis: same or different? A real-world study with emphasis on comorbidities. Clin Exp Rheumatol (2022) 40:1267–72. [doi: 10.55563/clinexprheumatol/8zn9z8](http://10.55563/clinexprheumatol/8zn9z8)

S8 Gubar ЕЕ, Korotaeva ТV, Dubinina ТV, Vorobyova LD, Tremaskina PO, Agafonova EM, et al. Comparative characteristics of axial spondyloarthritis and psoriatic arthritis with axial involvement. Rheumatology Science and Practice (2024) 62:168–75. [doi: 10.47360/1995-4484-2024-168-175](http://10.47360/1995-4484-2024-168-175)

S9 Haroon M, Ahmad M, Baig MN, Mason O, Rice J, FitzGerald O. Inflammatory back pain in psoriatic arthritis is significantly more responsive to corticosteroids compared to back pain in ankylosing spondylitis: a prospective, open-labelled, controlled pilot study. Arthritis Res Ther (2018) 20:73. [doi: 10.1186/s13075-018-1565-4](http://10.1186/s13075-018-1565-4)

S10 Jadon DR, Sengupta R, Nightingale A, Lu H, Dunphy J, Green A, et al. Serum bone-turnover biomarkers are associated with the occurrence of peripheral and axial arthritis in psoriatic disease: a prospective cross-sectional comparative study. Arthritis Res Ther (2017) 19:210. [doi: 10.1186/s13075-017-1417-7](http://10.1186/s13075-017-1417-7)

S11 Kavanaugh A, Baraliakos X, Gao S, Chen W, Sweet K, Chakravarty SD, et al. Genetic and Molecular Distinctions Between Axial Psoriatic Arthritis and Radiographic Axial Spondyloarthritis: Post Hoc Analyses from Four Phase 3 Clinical Trials. Adv Ther (2023) 40:2439–56. [doi: 10.1007/s12325-023-02475-4](http://10.1007/s12325-023-02475-4)

S12 Kwok TSH, Sutton M, Pereira D, Cook RJ, Chandran V, Haroon N, et al. Isolated axial disease in psoriatic arthritis and ankylosing spondylitis with psoriasis. Ann Rheum Dis (2022) 81:1678–84. [doi: 10.1136/ard-2022-222537](http://10.1136/ard-2022-222537)

S13 Michelena X, Lopez-Medina C, Erra A, Juanola X, Font-Ugalde P, Collantes E, et al. Characterising the axial phenotype of psoriatic arthritis: a study comparing axial psoriatic arthritis and ankylosing spondylitis with psoriasis from the REGISPONSER registry. RMD open (2022) 8. [doi: 10.1136/rmdopen-2022-002513](http://10.1136/rmdopen-2022-002513)

S14 Pereira GC, Machado NP, Gomes AF, Assad RL, Carneiro FH, Azevedo VF. Sacroiliac and spine imaging in spondyloarthritis: Does phenotype or sex matter? Adv Rheumatol (2024) 64:68. [doi: 10.1186/s42358-024-00411-w](http://10.1186/s42358-024-00411-w)

S15 Yanushonite AA, Korsakova YL, Korotaeva TV, Gubar EE, Loginova EYu, Urumova MM, et al. Comparative analysis of the frequency of comorbid diseases in axial psoriatic arthritis and other variants of axial spondyloarthritis. Data from a hospital cohort. Mod Rheumatol J (2024) 18:22–30. [doi: 10.14412/1996-7012-2024-5-22-30](http://10.14412/1996-7012-2024-5-22-30)

- 1. **Studies Included in the Systematic Review Without Meta-Analysis**

S16 Fernandez-Sueiro JL, Willisch A, Pertega-Diaz S, Tasende JA, Fernández‐Lopez C, Galdo F, et al. Evaluation of ankylosing spondylitis spinal mobility measurements in the assessment of spinal involvement in psoriatic arthritis. Arthritis Rheum (2009) 61:386–92. [doi: 10.1002/art.24280](http://10.1002/art.24280)

**3. Data Extraction**

**3.1 Subgroup Data Combination**

During the extraction of raw data for continuous variables, it was found that the group design in some studies differed from that of our study, thus requiring subgroup pooling. Sach as Table 2 of the fifth study.

Therefore, we applied the following formula to combine the subgroup data.

Formula: Let the sample size of subgroup A be N1, with mean M1 and standard deviation SD; the sample size of subgroup B be N2, with mean M2 and standard deviation SD2. Therefore, the combined result was:

N = N1 + N2

M = (N1 * M1 + N2 * M2) / (N1 + N2),


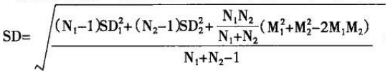


**3.2 Raw Outcome Data Extraction**

The data extraction process proceeded relatively smoothly. However, some studies showed discrepancies between the total number of survey participants and the number of participants measured for individual indicators. Therefore, during each meta-analysis, data were extracted based on the actual number of participants measured. For example, refer to Table 1 in the third study.

**3.2.1 BASDAI**

| **authors** | **year** | **axpsa_mean** | **axpsa_sd** | **n_axpsa** | **axspa_mean** | **axspa_sd** | **n_axspa** |
| --- | --- | --- | --- | --- | --- | --- | --- |
| **Diego Benavent** | 2021 | 4.7 | 2.5 | 367 | 3.7 | 2.4 | 2651 |
| **Diego Benavent** | 2020 | 5.23 | 2.1 | 65 | 6.1 | 4.5 | 287 |
| **Adrian Ciurea** | 2022 | 5.1 | 2.3 | 172 | 4.6 | 2.3 | 3212 |
| **Ran Cui** | 2022 | 1.8 | 1.85 | 68 | 2 | 0.2 | 446 |
| **Joy Feld** | 2020 | 3.5 | 2.2 | 477 | 3.9 | 2.1 | 766 |
| **Jose´ Luis Fernández-Sueiro** | 2010 | 2.7 | 1.9 | 46 | 2.8 | 1.9 | 103 |
| **G.E. Fragoulis** | 2022 | 3.37 | 1.93 | 79 | 2.96 | 1.95 | 129 |
| **E. E. Gubar** | 2025 | 5 | 2.2 | 55 | 3 | 2.2 | 45 |
| **Muhammad Haroon** | 2018 | 5.95 | 1.24 | 15 | 5.86 | 1.32 | 15 |
| **Arthur Kavanaugh** | 2023 | 6.4 | 1.7 | 190 | 7.4 | 1.3 | 323 |
| **Timothy S H Kwok** | 2022 | 1.97 | 1.03 | 32 | 4.23 | 2.59 | 82 |
| **Xabier Michelena** | 2022 | 4.2 | 3.1 | 109 | 4.1 | 3 | 127 |
| **Gabriel Caetano Pereira** | 2024 | 4.8 | 2.1 | 32 | 4.4 | 2 | 62 |
| **Yanushonite A.A.** | 2024 | 5.6 | 1.3 | 30 | 5.4 | 1.1 | 30 |

**3.2.2 ASDAS-crp**

| **authors** | **year** | **axpsa_mean** | **axpsa_sd** | **n_axpsa** | **axspa_mean** | **axspa_sd** | **n_axspa** |
| --- | --- | --- | --- | --- | --- | --- | --- |
| **Diego Benavent** | 2021 | 2.7 | 1.1 | 367 | 2.5 | 1.1 | 2651 |
| **Diego Benavent** | 2020 | 3.1 | 1 | 65 | 3.3 | 1 | 287 |
| **Adrian Ciurea** | 2022 | 2.9 | 1.1 | 32 | 2.8 | 1.1 | 2972 |
| **Ran Cui** | 2022 | 1.93 | 1.1 | 68 | 2.32 | 1 | 446 |
| **G.E. Fragoulis** | 2022 | 2.05 | 0.77 | 79 | 2.08 | 0.83 | 129 |
| **E. E. Gubar** | 2025 | 2.46 | 0.76 | 55 | 1.76 | 0.91 | 45 |
| **Deepak R Jadon** | 2017 | 2.2 | 1 | 117 | 2.1 | 0.9 | 157 |
| **Arthur Kavanaugh** | 2023 | 6.7 | 1.1 | 190 | 4.3 | 0.8 | 323 |
| **Xabier Michelena** | 2022 | 2.4 | 1.3 | 109 | 2.7 | 1.2 | 127 |
| **Gabriel Caetano Pereira** | 2024 | 3.2 | 1 | 32 | 2.7 | 1.1 | 62 |

**3.2.3 BASFI**

| **authors** | **year** | **axpsa_mean** | **axpsa_sd** | **n_axpsa** | **axspa_mean** | **axspa_sd** | **n_axspa** |
| --- | --- | --- | --- | --- | --- | --- | --- |
| **Diego Benavent** | 2021 | 3.6 | 2.7 | 367 | 3 | 2.6 | 2651 |
| **Adrian Ciurea** | 2022 | 3.5 | 2.7 | 176 | 3 | 2.5 | 3165 |
| **Ran Cui** | 2022 | 0.25 | 1.4 | 68 | 0.6 | 1.5 | 446 |
| **Jose´ Luis Fernández-Sueiro** | 2010 | 2.8 | 2.2 | 46 | 3.3 | 2.6 | 103 |
| **E. E. Gubar** | 2025 | 3.7 | 3.8 | 55 | 1.1 | 3.3 | 45 |
| **Muhammad Haroon** | 2018 | 6.4 | 1.52 | 15 | 6.06 | 1.49 | 15 |
| **Xabier Michelena** | 2022 | 3.2 | 3.2 | 109 | 3.8 | 3.6 | 127 |
| **Gabriel Caetano Pereira** | 2024 | 8.1 | 14.4 | 32 | 4.3 | 2.7 | 62 |

**3.2.4 CRP**

| **authors** | **year** | **axpsa_mean** | **axpsa_sd** | **n_axpsa** | **axspa_mean** | **axspa_sd** | **n_axspa** |
| --- | --- | --- | --- | --- | --- | --- | --- |
| **Diego Benavent** | 2020 | 11.1 | 12.7 | 65 | 12.6 | 18.9 | 287 |
| **Adrian Ciurea** | 2022 | 5 | 4.5 | 1022 | 4 | 5.7 | 4025 |
| **Ran Cui** | 2022 | 3.5 | 12.7 | 68 | 6.8 | 11.9 | 446 |
| **E. E. Gubar** | 2025 | 6.8 | 10.7 | 55 | 4.5 | 6.4 | 45 |
| **Muhammad Haroon** | 2018 | 9 | 4.74 | 15 | 7.27 | 3.61 | 15 |
| **Deepak R Jadon** | 2017 | 3 | 3.7 | 117 | 4 | 6.7 | 157 |
| **Arthur Kavanaugh** | 2023 | 2.8 | 3.4 | 190 | 2.2 | 2.6 | 323 |
| **Xabier Michelena** | 2022 | 9.3 | 16 | 109 | 9.3 | 15.9 | 127 |
| **Yanushonite A.A.** | 2024 | 20.5 | 13.3 | 30 | 13.6 | 8.2 | 30 |

**3.2.5 HLA-B27**

| **authors** | **year** | **axpsa_event** | **axpsa_total** | **axspa_event** | **axspa_total** |
| --- | --- | --- | --- | --- | --- |
| **Diego Benavent** | 2021 | 54 | 182 | 1674 | 2126 |
| **Diego Benavent** | 2020 | 16 | 47 | 204 | 281 |
| **Adrian Ciurea** | 2022 | 150 | 674 | 2579 | 3997 |
| **Ran Cui** | 2022 | 22 | 61 | 378 | 446 |
| **Joy Feld** | 2020 | 91 | 477 | 584 | 766 |
| **G.E. Fragoulis** | 2022 | 8 | 38 | 72 | 89 |
| **E. E. Gubar** | 2025 | 14 | 55 | 40 | 45 |
| **Deepak R Jadon** | 2017 | 47 | 117 | 140 | 157 |
| **Timothy S H Kwok** | 2022 | 11 | 32 | 62 | 82 |
| **Xabier Michelena** | 2022 | 26 | 86 | 62 | 93 |
| **Gabriel Caetano Pereira** | 2024 | 8 | 32 | 43 | 62 |

3.3 Covariate Definition and Extraction
A total of seven covariates were extracted for this meta-regression. They include the overall study characteristics—mainly the definition of axial involvement in ax-PsA, control group type, and sample size—as well as differences in population characteristics between the two groups, mainly sex, age, disease duration, and peripheral arthritis. The specific extraction criteria are as follows:

(1) Definition of axial involvement in ax-PsA: Among the 15 included studies, three diagnostic criteria were used: (i) the 1984 New York criteria, with radiographic sacroiliitis graded as bilateral ≥2 or unilateral ≥3; (ii) the imaging arm of the ASAS criteria for ax-SpA diagnosis, requiring one clinical feature of SpA plus active inflammation on sacroiliac MRI or radiographic sacroiliitis; (iii) a diagnosis of axial involvement made by rheumatologists based on the above two criteria combined with patient evaluation. Based on these categories, the definition of axial involvement in ax-PsA was converted into two dummy variables—the modified New York (mNY) criteria and the ASAS criteria—for inclusion in the meta-regression.

(2) Control group type (Group-AS): Some control groups explicitly defined ax-SpA as AS. Since AS is a subtype of ax-SpA, only the AS group was included as a covariate.

(3) Sample size (SS): The total number of participants (ax-PsA and ax-SpA) involved in the clinical studies.

(4) Sex: The sex characteristic in this study is the difference between the proportion of males in ax‑SpA and the proportion of males in ax‑PsA.

(5) Age: In the included studies, current age and age at diagnosis are usually reported. Considering that the assessment of relevant clinical features corresponds to current age, the age variable in this study is the difference between the mean current age of ax‑SpA patients and the mean current age of ax‑PsA patients. If not specified in detail, the age difference as reported in the original article was used.

(6) Disease duration (DD): Regarding disease duration, the included studies reported the duration from onset of any symptoms, the duration after the appearance of inflammatory back pain, and the delayed diagnosis duration. Given that the core focus of this study is to compare the difference in axial involvement between ax‑SpA and ax‑PsA, and that the main scores are ax‑SpA‑related indices, we selected the difference in disease duration after the appearance of inflammatory back pain between ax‑SpA and ax‑PsA. If not specified in detail, the disease duration difference as reported in the original article was used.

(7) Peripheral arthritis (PA): Regarding peripheral arthritis, there were substantial missing data in the included studies. In the few studies that reported peripheral arthritis, the forms of reporting varied considerably. Therefore, to extract the peripheral arthritis characteristics of each included study as comprehensively as possible, this covariate was defined as the difference between the proportion of ax‑SpA patients with peripheral arthritis symptoms and the proportion of ax‑PsA patients with peripheral arthritis symptoms.

**4 Exploration of Heterogeneity**

**4.1 BASDAI**

First, we performed sensitivity analysis to identify potential sources of heterogeneity; no obvious outlier data were identified.


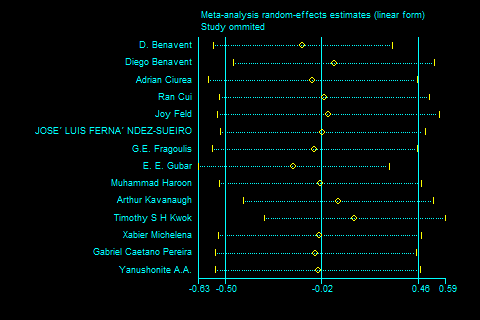


Subsequently, meta-regression was conducted on each of the eight covariates, and all results were statistically non-significant.

| Covariate | Beta | SE | CI_LB | CI_UB | Z | P_Value |
| --- | --- | --- | --- | --- | --- | --- |
| Group-AS | -0.966058767 | 0.472403996866479 | -1.995338656 | 0.0632211223006101 | -2.044984321 | 0.0634307201923099 |
| mNY | -0.324176171 | 0.559403382918006 | -1.543011438 | 0.894659096992049 | -0.579503415 | 0.572968437145372 |
| ASAS | 0.408728594251623 | 0.668011251940589 | -1.046742892 | 1.86420008034185 | 0.611858846784777 | 0.552047737810887 |
| SS | 0.000235191915936007 | 0.000239955119383651 | -0.000287625 | 0.000758009208593436 | 0.980149606893661 | 0.346360379922855 |
| Sex | -1.762631259 | 3.20575648191707 | -8.818453703 | 5.29319118427538 | -0.549833173 | 0.593421739077261 |
| Age | -0.077444883 | 0.0809556441895883 | -0.255627054 | 0.100737288974087 | -0.956633516 | 0.359304841484667 |
| DD | 0.035444009735467 | 0.207300736926182 | -0.433502837 | 0.504390856626376 | 0.170978696270088 | 0.868022761933072 |
| PA | -2.254989428 | 2.20650266809456 | -7.472539148 | 2.96256029227202 | -1.021974485 | 0.34080724827155 |

Although no covariate was found to be significantly associated in the meta‑regression, bubble plots and prediction interval plots were still generated for the continuous covariates. The results showed that both the 95% confidence intervals (CIs) and the 95% prediction intervals (PIs) crossed the midline of MD = 0, indicating that the meta-regression results remained stable.


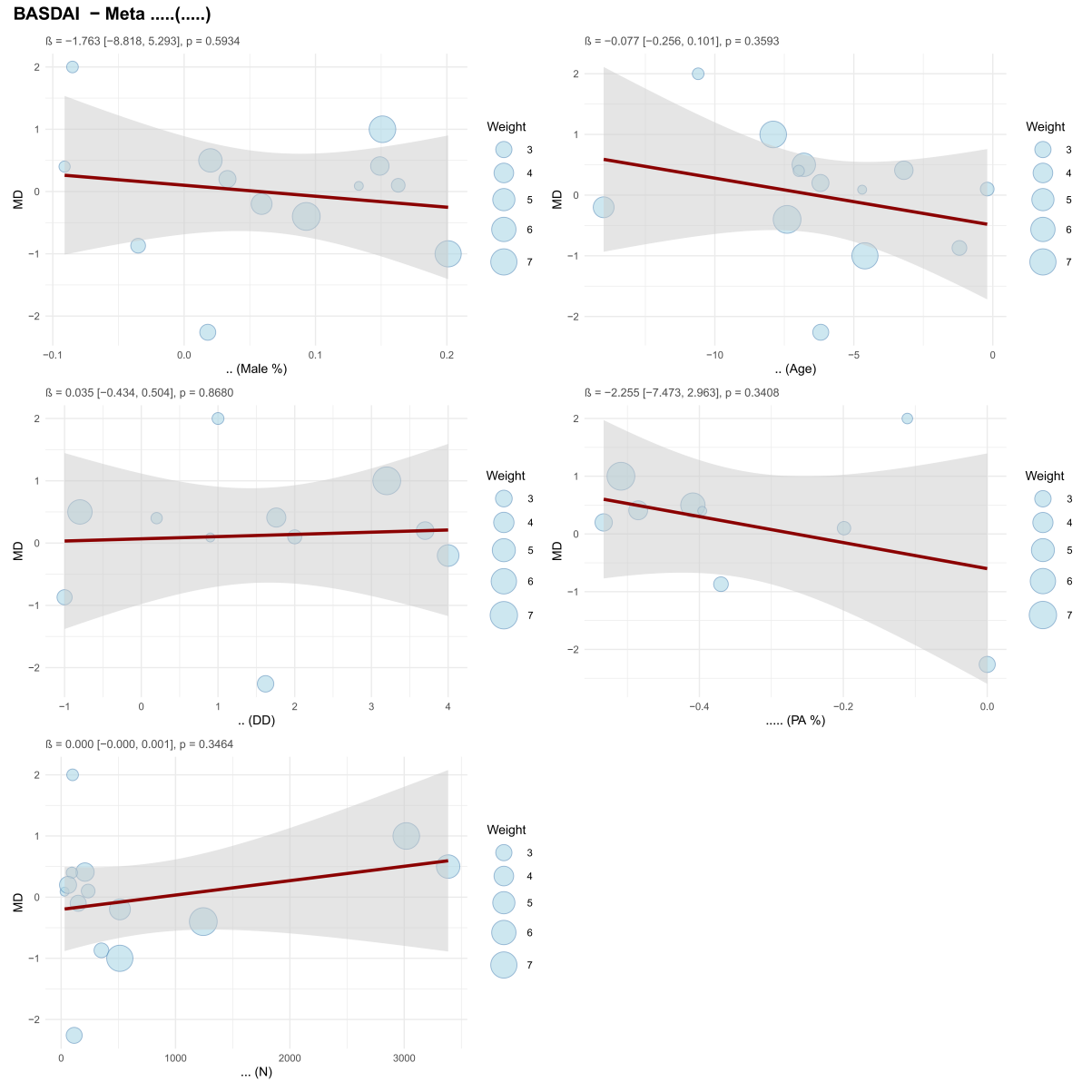

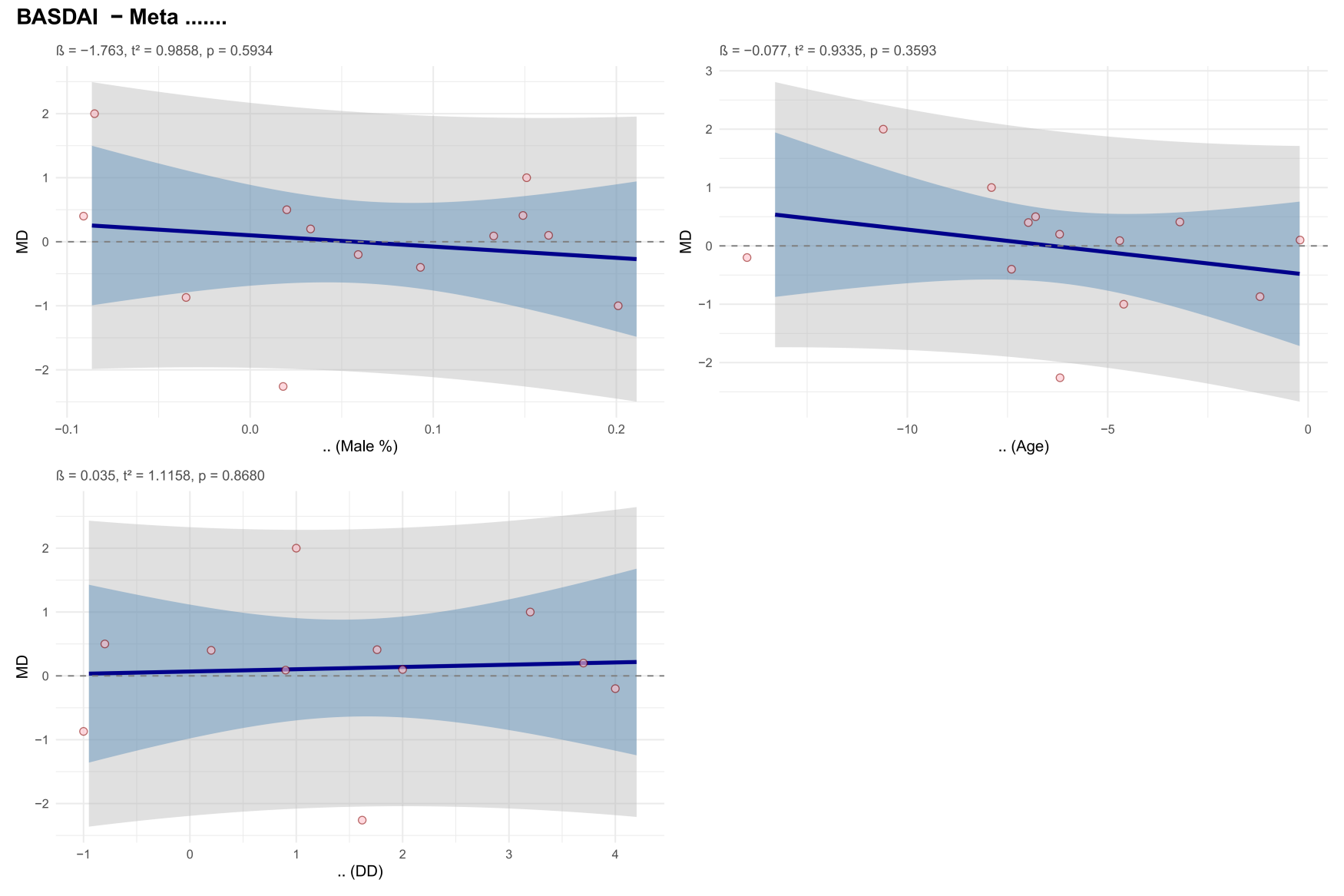


**4.2 ASDAS**

Potential sources of heterogeneity were explored through sensitivity analysis, and no significant outliers were identified.


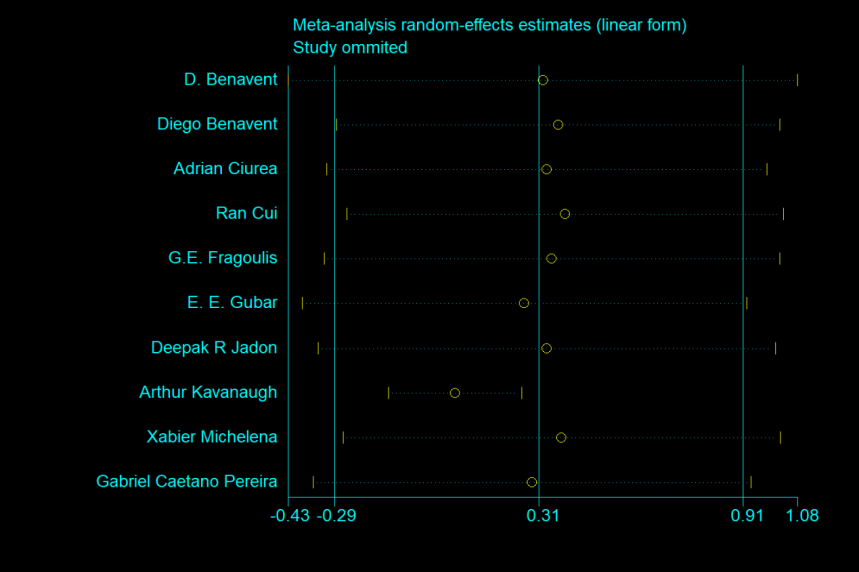


Meta-regression on the full set of eight covariates likewise showed no statistically significant findings.

| Covariate | Beta | SE | CI_LB | CI_UB | Z | P_Value |
| --- | --- | --- | --- | --- | --- | --- |
| Group-AS | 0.105021630664659 | 0.545265900149504 | -1.15236379 | 1.36240705119524 | 0.192606269043899 | 0.852066950628429 |
| mNY | -0.251641497 | 0.589421730703201 | -1.610850445 | 1.10756745179609 | -0.426929452 | 0.680688115361375 |
| ASAS | -0.102058907 | 0.685361549095536 | -1.682505474 | 1.47838765889966 | -0.148912508 | 0.885308025126207 |
| SS | -5.19E-05 | 0.000248055488286833 | -0.000623918 | 0.00052011583842263 | -0.20923199 | 0.839496773280732 |
| Sex | 1.52620665614339 | 2.62790550185388 | -4.533754298 | 7.58616761034421 | 0.580769230501901 | 0.577385866223703 |
| Age | -0.001073486 | 0.0686859784478122 | -0.159463637 | 0.157316664101862 | -0.015628899 | 0.987913193573197 |
| DD | -0.039713618 | 0.0678297290527904 | -0.20010544 | 0.120678204468884 | -0.585489849 | 0.576598417751743 |
| PA | 0.550159454416974 | 1.0442844005921 | -2.134259057 | 3.23457796581738 | 0.526829141663937 | 0.620839666540851 |

Likewise, bubble plots and prediction interval plots were generated for the continuous covariates. The 95% confidence intervals and 95% prediction intervals all crossed the midline of MD = 0, indicating that the meta‑regression results remained stable.


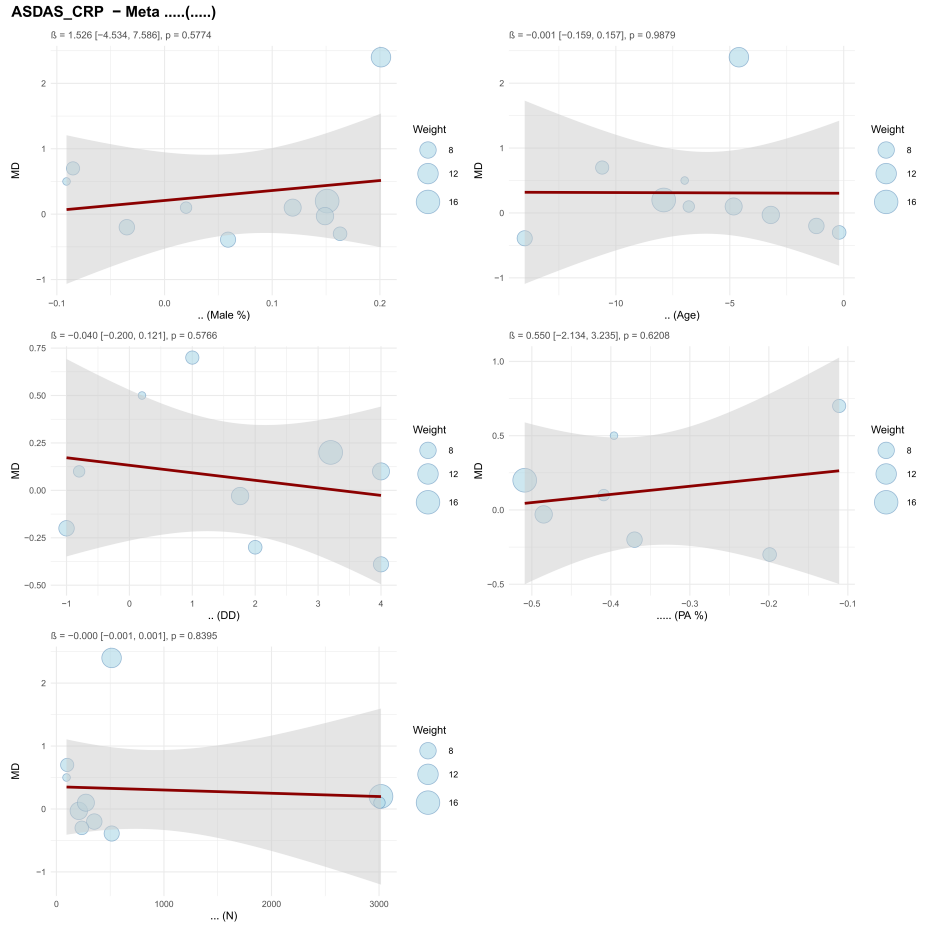


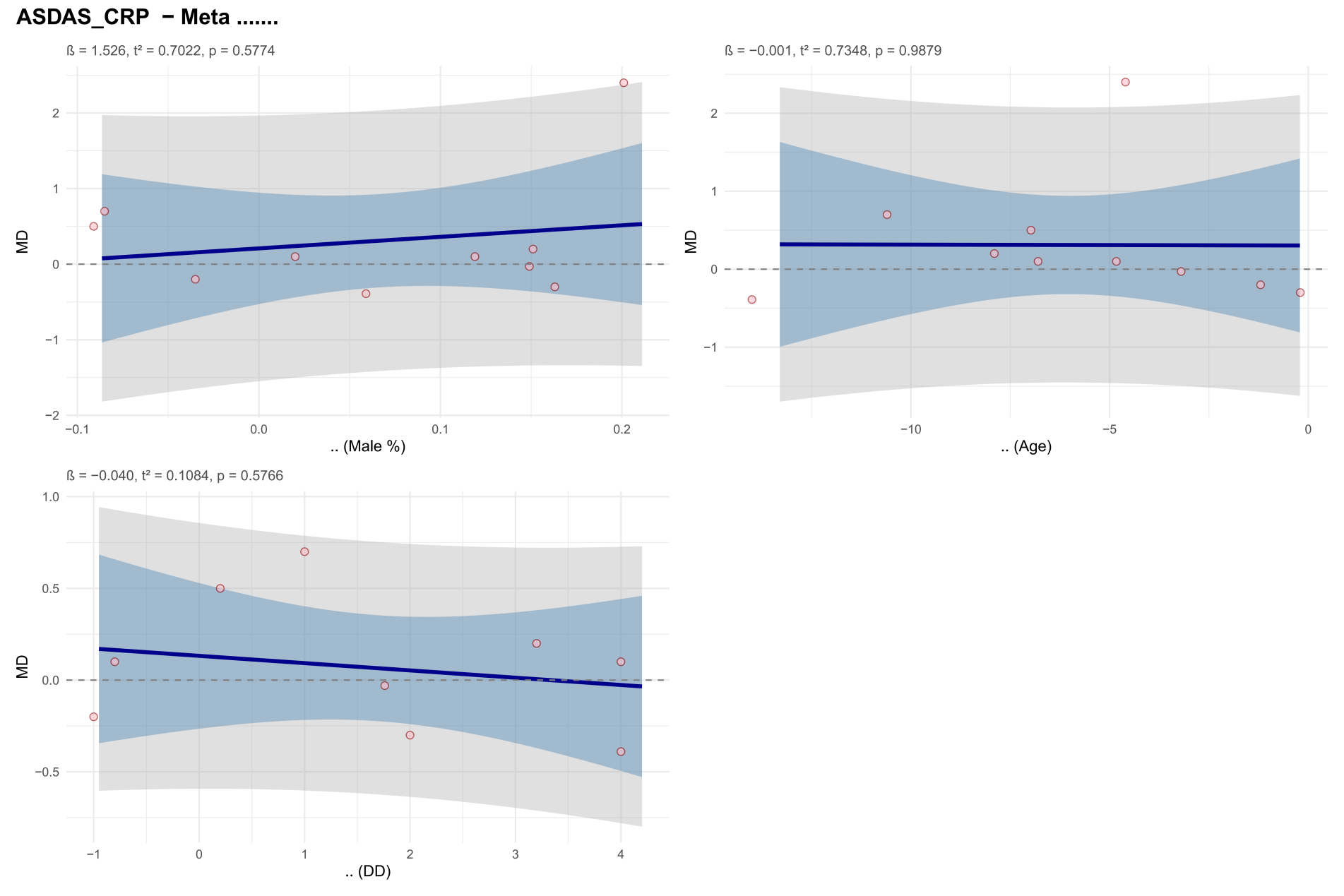


**4.3 BASFI**

Sensitivity analysis was conducted to investigate heterogeneity, and no significant outliers were identified.


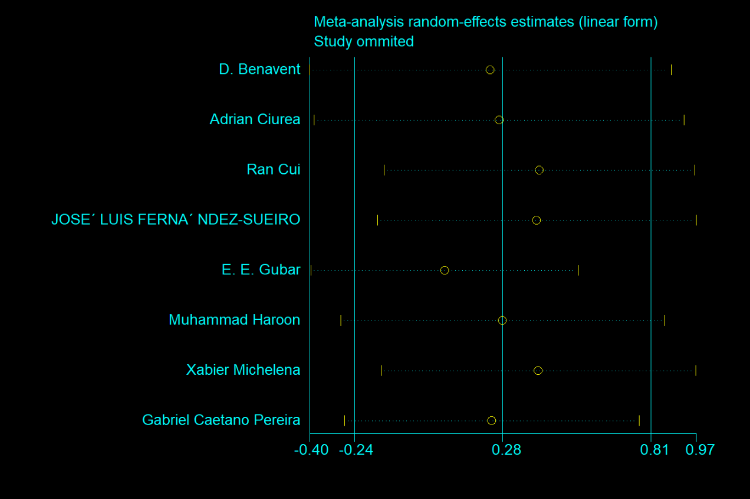


Meta-regression revealed that Group-AS was the main source of heterogeneity, as shown in the table below.

| Covariate | Beta | SE | CI_LB | CI_UB | Z | P_Value |
| --- | --- | --- | --- | --- | --- | --- |
| Group-AS | -0.977748447 | 0.268889631578043 | -1.635697673 | -0.31979922 | -3.63624451 | 0.0108836077338434 |
| mNY | 0.0174194452746025 | 0.866038809210176 | -2.101701181 | 2.13654007108246 | 0.0201139314882308 | 0.984604689558142 |
| ASAS | 0.521010256915389 | 1.19078483245836 | -2.392735262 | 3.43475577562143 | 0.437535180759542 | 0.677033414812461 |
| SS | 6.11139090606297e-05 | 0.000303248022033551 | -0.000680907 | 0.000803135088010797 | 0.201531105300559 | 0.846942128454548 |
| Sex | -8.573339629 | 3.85610305993492 | -18.48576811 | 1.33908885338479 | -2.223317037 | 0.0768073138441931 |
| Age | -0.059556046 | 0.108180149981444 | -0.337641974 | 0.218529882580076 | -0.550526561 | 0.605649636798511 |
| DD | -0.238904349 | 0.263556904272875 | -0.916398939 | 0.438590242096548 | -0.906462114 | 0.406253644041398 |
| PA | 1.71114115598095 | 4.73148770273175 | -13.3465644 | 16.7688467140349 | 0.36164971008865 | 0.741585147212777 |

For this indicator, the bubble plots and prediction interval plots yielded results consistent with the meta-regression, with no additional findings.


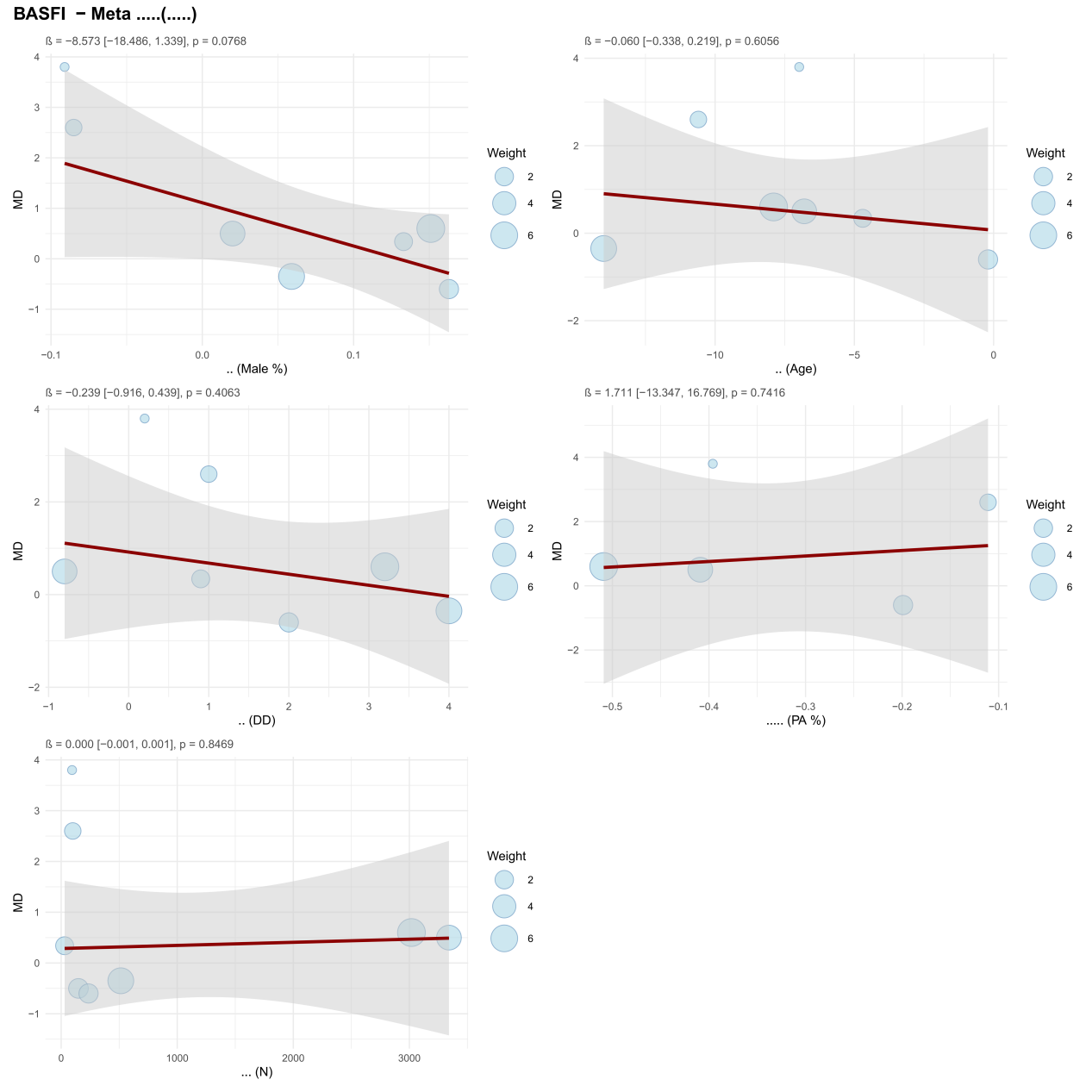


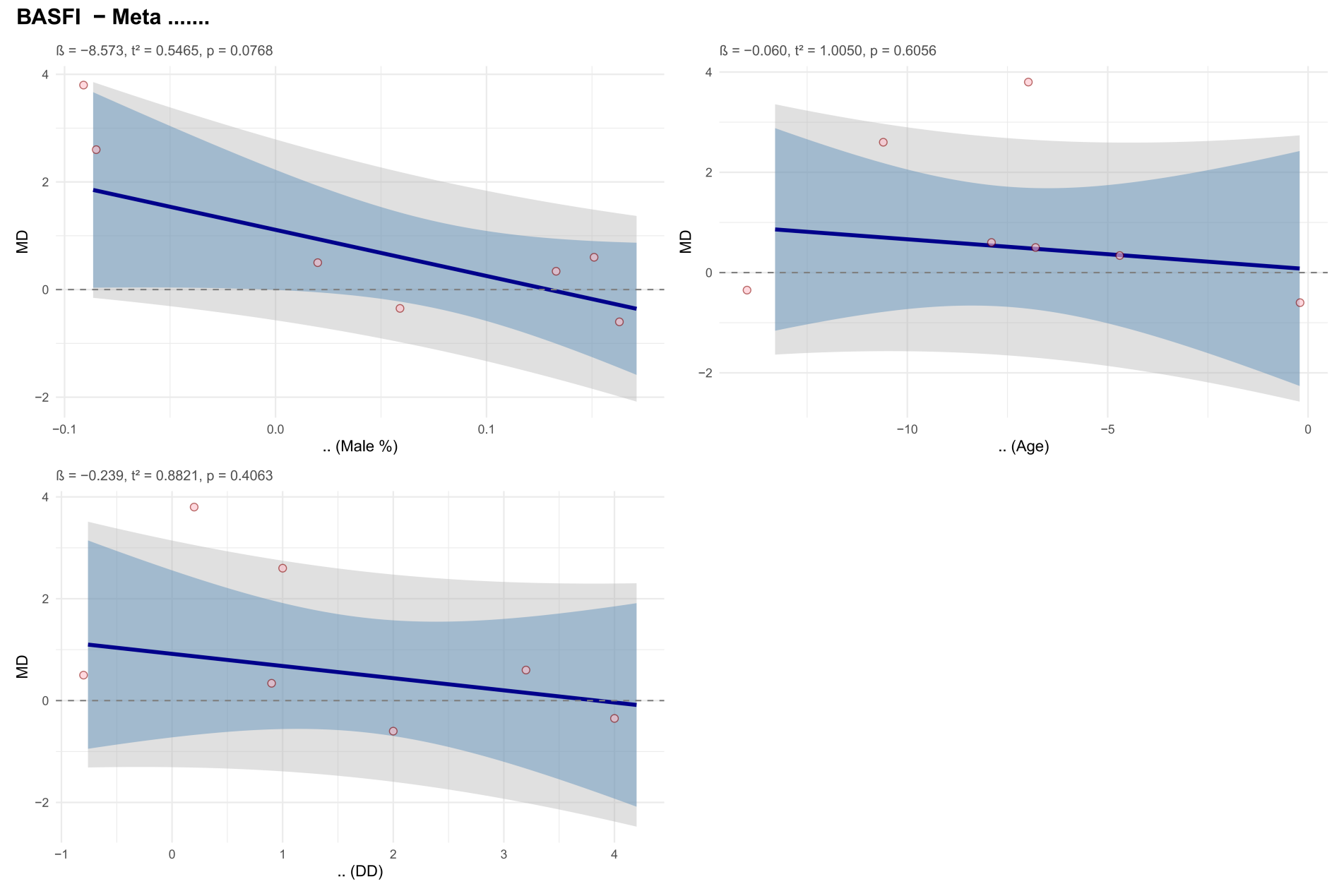


**4.4 CRP**

Sensitivity analysis revealed no articles with significant heterogeneity.


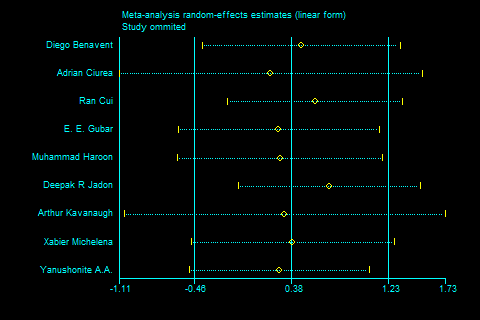


The meta-regression results were all non-significant.

| Covariate | Beta | SE | CI_LB | CI_UB | Z | P_Value |
| --- | --- | --- | --- | --- | --- | --- |
| Group-AS | -1.572359927 | 1.29832049504813 | -4.642400056 | 1.49768020226771 | -1.211072253 | 0.265163250760446 |
| mNY | -1.79584001 | 0.82041022035235 | -3.735801913 | 0.144121893188287 | -2.188953728 | 0.0647787505137763 |
| ASAS | 1.48771649019642 | 2.50146889081319 | -4.427317514 | 7.4027504940182 | 0.594737154501563 | 0.570734934922963 |
| SS | 0.000105741152899354 | 0.000405275602429665 | -0.000852583 | 0.00106406567098342 | 0.260911716040704 | 0.801669929664767 |
| Sex | -2.911079213 | 8.49160462070278 | -22.99053343 | 17.1683750080638 | -0.342818506 | 0.741799297307605 |
| Age | 0.0702434575806279 | 0.216300610361981 | -0.441226211 | 0.581713126476889 | 0.324749234239676 | 0.754860555500331 |
| DD | -0.250042688 | 0.453208462933504 | -1.359003847 | 0.8589184706821 | -0.551716724 | 0.601070749493506 |
| PA | -4.391351444 | 9.34562555373649 | -34.13330296 | 25.3506000705272 | -0.469883093 | 0.67048490586741 |

For CRP, the bubble plot and prediction interval plot revealed no further findings.


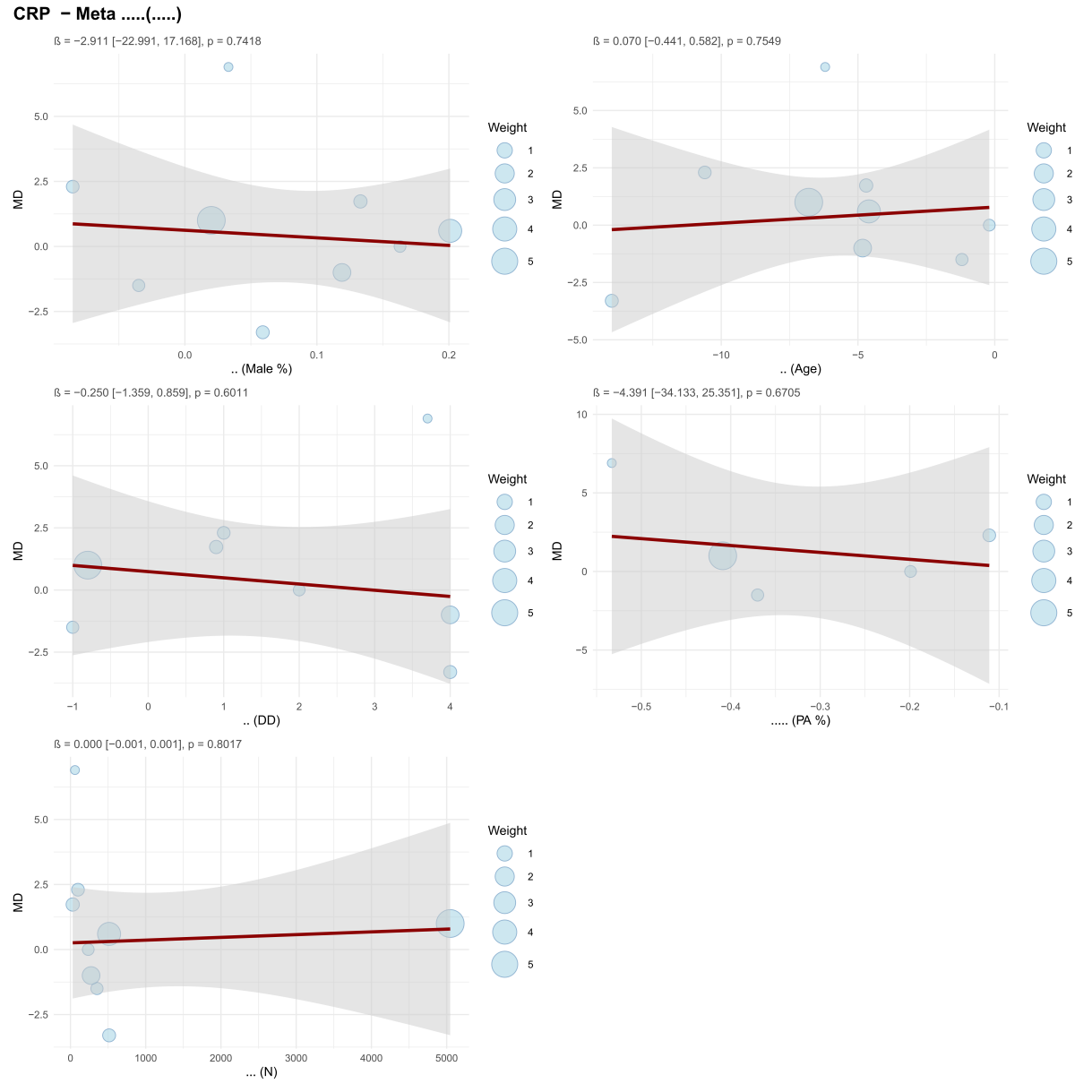


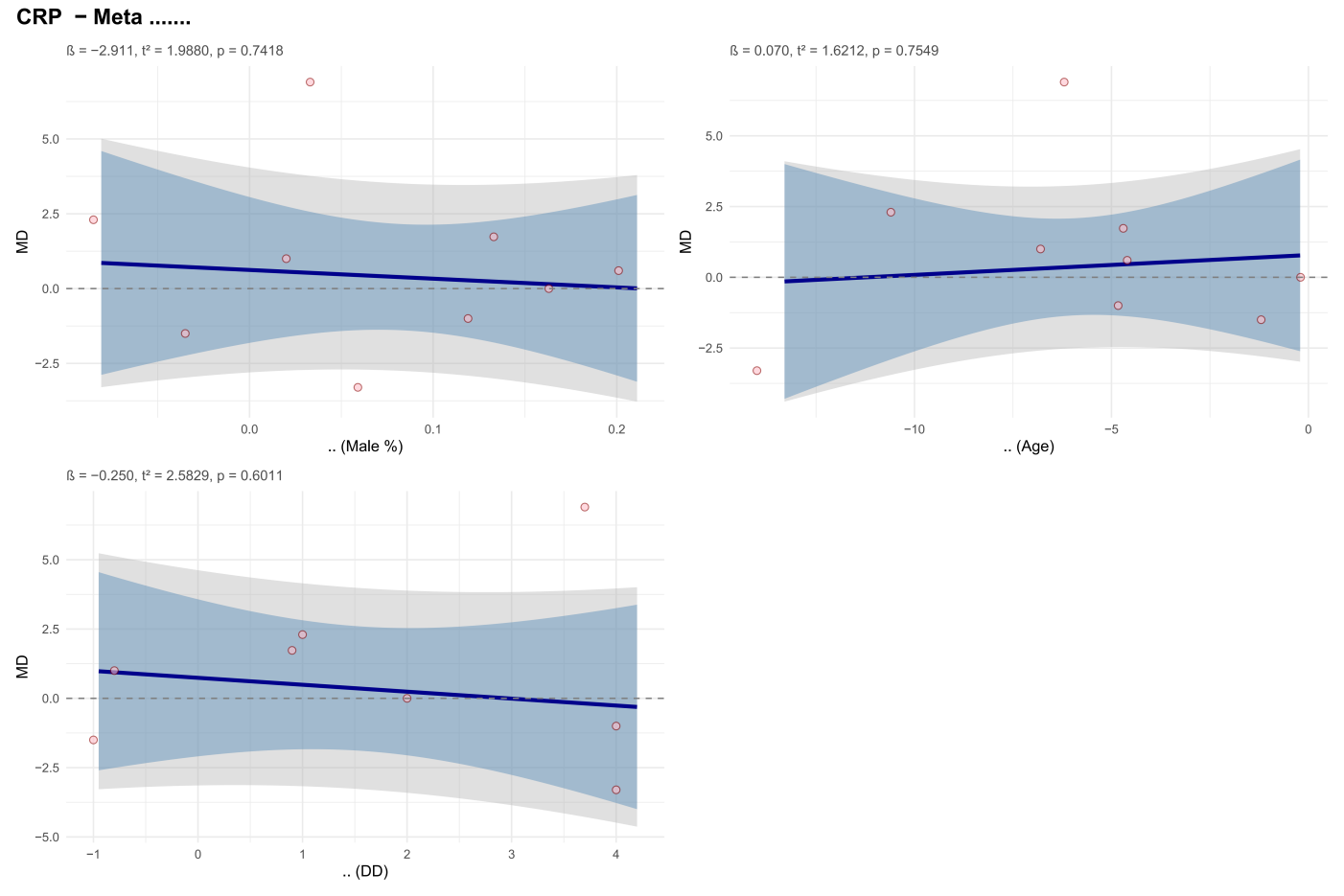


**4.5 HLA-B27**

As the data involved dichotomous variables, we first assessed heterogeneity using L'Abbé plots and radial plots, with no studies showing significant outliers identified. Subsequently, sensitivity analysis was performed, and again no abnormal results were found.


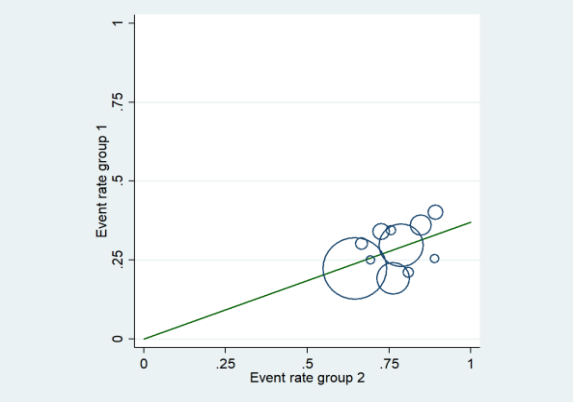

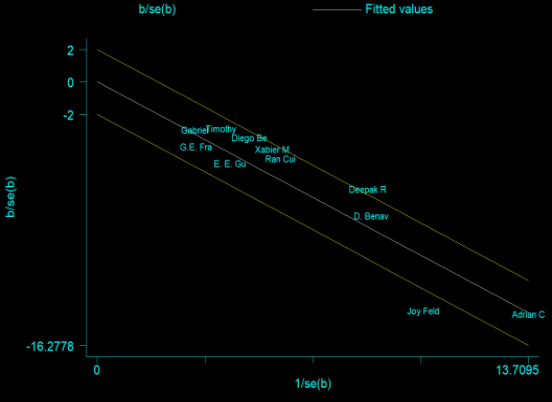

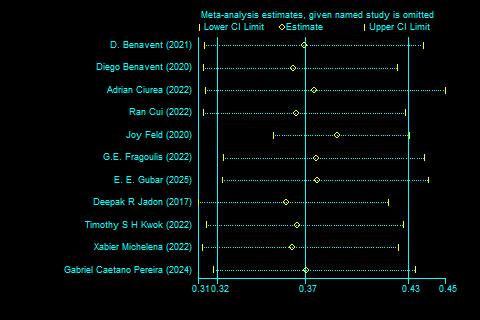


The results of the meta-regression were non-significant.

| Covariate | Beta | SE | CI_LB | CI_UB | Z | P_Value |
| --- | --- | --- | --- | --- | --- | --- |
| Group-AS | 0.0170479959171795 | 0.146727755966322 | -0.314873248 | 0.348969240057701 | 0.116187941435515 | 0.910054857206775 |
| mNY | -0.06363539 | 0.144884479039547 | -0.391386852 | 0.264116071882438 | -0.43921468 | 0.670862098572696 |
| ASAS | -0.197061321 | 0.252285764184488 | -0.767771369 | 0.373648727809582 | -0.781103608 | 0.454796185465959 |
| SS | -3.14E-05 | 4.52823635388082e-05 | -0.00013388 | 7.09917244677792e-05 | -0.694400559 | 0.50495961997245 |
| Sex | 0.186115053962537 | 0.919860994350757 | -1.894755083 | 2.26698519111178 | 0.202329542295571 | 0.844158854300108 |
| Age | 0.0170828769657472 | 0.0194228943677755 | -0.026854763 | 0.0610205165820834 | 0.879522724176962 | 0.401990898171041 |
| DD | 0.036710213669872 | 0.0206273477978167 | -0.010856536 | 0.0842769629899319 | 1.77968656124359 | 0.113004492098818 |
| PA | 0.290092079069803 | 0.405760477450356 | -0.702768042 | 1.28295220006932 | 0.714934290526842 | 0.501507188081972 |

For HLA‑B27 positivity rate, the bubble plot and prediction interval plot showed remarkably stable results, with the PI and CI almost completely overlapping when DD was used as the covariate.


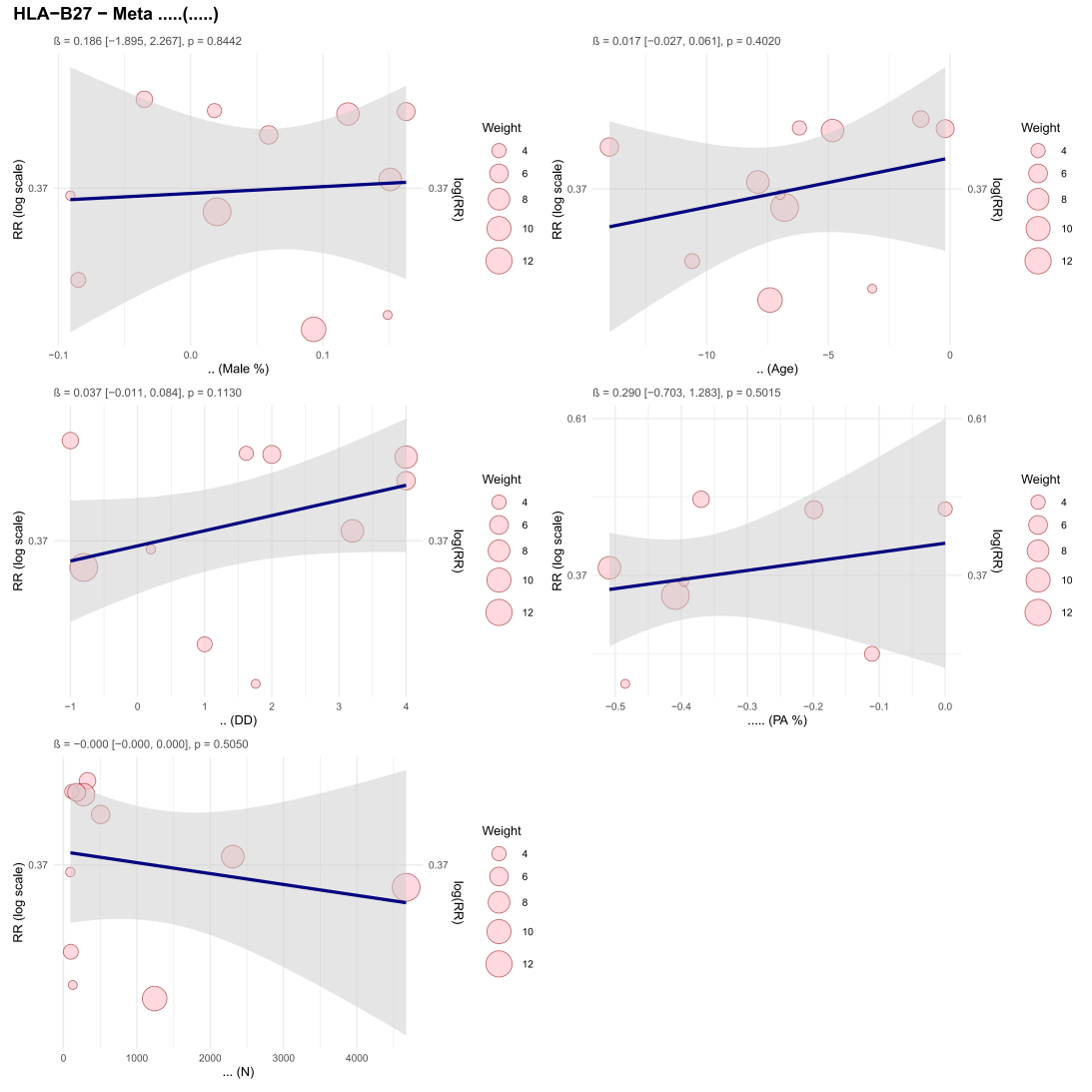


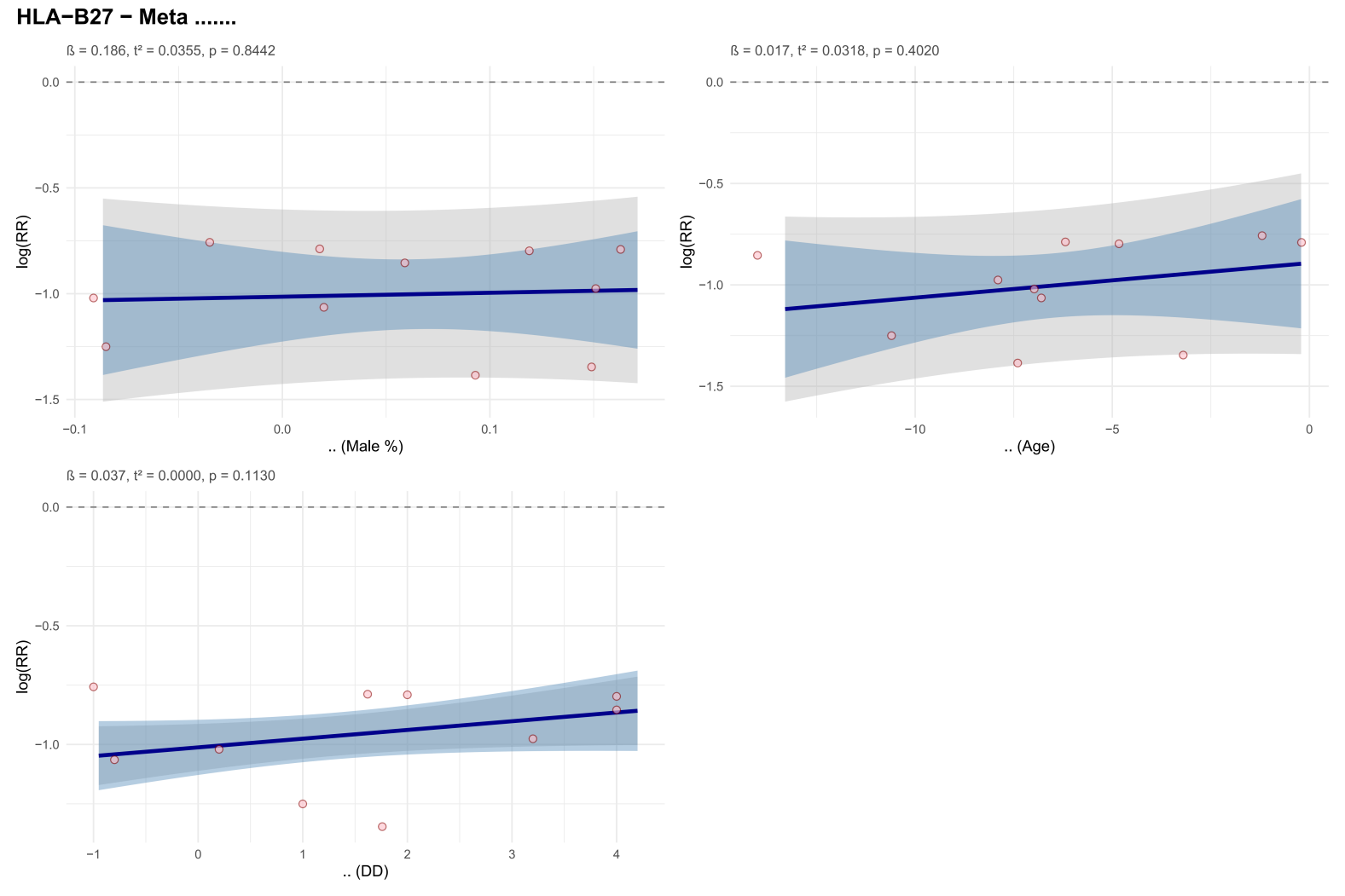


1. **GRADE Quality of Evidence Assessment**

**5.1 Question 1: Ax-PsA Compared to ax-SpA for Whether Disease Activity is Similar Between the Two Disease Subtypes?**

| **Certainty assessment** | | | | | | | **№ of patients** | | **Effect** | | **Certainty** | **Importance** |
| --- | --- | --- | --- | --- | --- | --- | --- | --- | --- | --- | --- | --- |
| **№ of studies** | **Study design** | **Risk of bias** | **Inconsistency** | **Indirectness** | **Imprecision** | **Other considerations** | **ax-PsA** | **ax-SpA** | **Relative (95% CI)** | **Absolute (95% CI)** |  |  |
| **Bath Ankylosing Spondylitis Disease Activity Index (assessed with: rhumatologue; Scale from: 0 to 10)** | | | | | | | | | | | | |
| 14 | non-randomised studies | not serious | very serious^a^ | not serious | not serious | none | 1737 | 8278 | - | MD **0.02 lower** (0.5 lower to 0.46 higher) | ⨁◯◯◯  Very low^a^ | CRITICAL |
| **Ankylosing Spondylitis Disease Activity Score (assessed with: rhumatologue; Scale from: 0.5 to 6.0)** | | | | | | | | | | | | |
| 10 | non-randomised studies | not serious | very serious^a^ | not serious | not serious | none | 1114 | 7199 | - | MD **0.31 higher** (0.29 lower to 0.91 higher) | ⨁◯◯◯  Very low^a^ | CRITICAL |

**5.2 Question 2: Ax-PsA Compared to ax-SpA for Whether the Physical Function Is Similar Between the Two Groups?**

| **Certainty assessment** | | | | | | | **№ of patients** | | **Effect** | | **Certainty** | **Importance** |
| --- | --- | --- | --- | --- | --- | --- | --- | --- | --- | --- | --- | --- |
| **№ of studies** | **Study design** | **Risk of bias** | **Inconsistency** | **Indirectness** | **Imprecision** | **Other considerations** | **ax-PsA** | **ax-SpA** | **Relative (95% CI)** | **Absolute (95% CI)** |  |  |
| **Bath Ankylosing Spondylitis Functional Index (assessed with: rhumatologue; Scale from: 0 to 10)** | | | | | | | | | | | | |
| 8 | non-randomised studies | not serious | serious^a^ | not serious | not serious | all plausible residual confounding would suggest spurious effect, while no effect was observed | 868 | 6614 | - | MD **0.28 higher** (0.24 lower to 0.81 higher) | ⨁⨁◯◯ Low^a^ | CRITICAL |

**5.3 Question 3: Ax-PsA Compared to ax-SpA for Whether the Systemic Inflammatory Markers are Similar Between the Two Groups**

| **Certainty assessment** | | | | | | | **№ of patients** | | **Effect** | | **Certainty** | **Importance** |
| --- | --- | --- | --- | --- | --- | --- | --- | --- | --- | --- | --- | --- |
| **№ of studies** | **Study design** | **Risk of bias** | **Inconsistency** | **Indirectness** | **Imprecision** | **Other considerations** | **ax-PsA** | **ax-SpA** | **Relative (95% CI)** | **Absolute (95% CI)** |  |  |
| **C reactive protein (assessed with: rhumatologue)** | | | | | | | | | | | | |
| 9 | non-randomised studies | not serious | serious^a^ | not serious | not serious | none | 1671 | 5455 | - | MD **0.38 mg/L higher** (0.46 lower to 1.23 higher) | ⨁◯◯◯  Very low^a^ | IMPORTANT |

**5.4 Question 4: Ax-PsA Compared to ax-SpA for Whether There Is a Difference in the HLA-B27 Positivity Rate Between the Two Groups?**

| **Certainty assessment** | | | | | | | **№ of patients** | | **Effect** | | **Certainty** | **Importance** |
| --- | --- | --- | --- | --- | --- | --- | --- | --- | --- | --- | --- | --- |
| **№ of studies** | **Study design** | **Risk of bias** | **Inconsistency** | **Indirectness** | **Imprecision** | **Other considerations** | **ax-PsA** | **ax-SpA** | **Relative (95% CI)** | **Absolute (95% CI)** |  |  |
| **HLA-B27 positivity rates (assessed with: rhumatologue)** | | | | | | | | | | | | |
| 11 | non-randomised studies | not serious | serious^a^ | not serious | not serious | none | 447/1801 (24.8%) | 5838/8411 (69.4%) | **RR** 0.37 (0.32 to 0.43) | **437 fewer per 1,000** (from 472 fewer to 396 fewer) | ⨁◯◯◯  Very low^a^ | CRITICAL |
|  |  |  |  |  |  |  |  | 80.0% |  | **504 fewer per 1,000** (from 544 fewer to 456 fewer) |  |  |
