# Supplementary material for: Differences in clinical features between axial psoriatic arthritis and axial spondyloarthritis: a systematic review and meta-analysis of observational studies
Source: Front Med (Lausanne). 2026 Jun 22;13:1856665. doi: 10.3389/fmed.2026.1856665 (PMC13333385; doi:10.3389/fmed.2026.1856665)
Supplement: Supplementary file 2 [file Data_Sheet_2.docx]

# =============================================================================

# Meta‑regression only: ax-PsA vs ax-SpA/AS

# Continuous outcomes (WMD) and binary outcome (RR)

# Knapp‑Hartung correction enabled (test = "knha")

# =============================================================================

# Load required packages

required_packages <- c("metafor", "dplyr", "data.table")

for (pkg in required_packages) {

if (!require(pkg, character.only = TRUE, quietly = TRUE)) {

install.packages(pkg, repos = "https://cloud.r-project.org")

library(pkg, character.only = TRUE)

}

}

# =============================================================================

# Working directory (absolute path)

# =============================================================================

work_dir <- "C:/Users/11638/Documents/meta_analysis"

if (!dir.exists(work_dir)) dir.create(work_dir, recursive = TRUE)

setwd(work_dir)

csv_dir <- file.path(work_dir, "csv_data")

if (!dir.exists(csv_dir)) stop("CSV data folder not found: ", csv_dir)

cat("Working directory:", getwd(), "\n")

cat("CSV data directory:", csv_dir, "\n")

# Required CSV files

required_csv <- c("BASDAI.csv", "ASDAS_crp.csv", "BASFI.csv", "crp.csv", "HLA_B27.csv")

missing <- required_csv[!file.exists(file.path(csv_dir, required_csv))]

if (length(missing) > 0) {

stop("Missing CSV file(s): ", paste(missing, collapse = ", "))

}

# Read raw data

basdai_raw <- read.csv(file.path(csv_dir, "BASDAI.csv"), stringsAsFactors = FALSE, fileEncoding = "UTF-8")

asdas_crp_raw <- read.csv(file.path(csv_dir, "ASDAS_crp.csv"), stringsAsFactors = FALSE, fileEncoding = "UTF-8")

basfi_raw <- read.csv(file.path(csv_dir, "BASFI.csv"), stringsAsFactors = FALSE, fileEncoding = "UTF-8")

crp_raw <- read.csv(file.path(csv_dir, "crp.csv"), stringsAsFactors = FALSE, fileEncoding = "UTF-8")

hla_raw <- read.csv(file.path(csv_dir, "HLA_B27.csv"), stringsAsFactors = FALSE, fileEncoding = "UTF-8")

# =============================================================================

# Data cleaning functions

# =============================================================================

clean_continuous <- function(df, outcome_name) {

df %>%

filter(!is.na(authors)) %>%

rename(study = authors, year = year,

mean_axpsa = axpsa_mean, sd_axpsa = axpsa_sd, n_axpsa = n_axpsa,

mean_axspa = axspa_mean, sd_axspa = axspa_sd, n_axspa = n_axspa,

population_c = population_c,

definition_axpsa = The.definition.of.axpsa,

sex = sex, age = age, dd = DD, pa = PA) %>%

mutate(outcome = outcome_name) %>%

mutate(across(where(is.character), ~na_if(., "NA"))) %>%

mutate(mean_axpsa = as.numeric(mean_axpsa), sd_axpsa = as.numeric(sd_axpsa),

n_axpsa = as.numeric(n_axpsa),

mean_axspa = as.numeric(mean_axspa), sd_axspa = as.numeric(sd_axspa),

n_axspa = as.numeric(n_axspa),

population_c = as.numeric(population_c),

definition_axpsa = as.numeric(definition_axpsa),

sex = as.numeric(sex), age = as.numeric(age),

dd = as.numeric(dd), pa = as.numeric(pa)) %>%

mutate(study_id = paste0(study, "_", year),

n_total = n_axpsa + n_axspa,

md = mean_axpsa - mean_axspa,

se = sqrt((sd_axpsa^2 / n_axpsa) + (sd_axspa^2 / n_axspa)),

vi = se^2,

ctrl_as = ifelse(population_c == 1, 1, 0),

ctrl_axspa = ifelse(population_c == 0, 1, 0),

def_mny = ifelse(definition_axpsa == 1, 1, 0),

def_asas = ifelse(definition_axpsa == 2, 1, 0),

def_other = ifelse(definition_axpsa == 3, 1, 0)) %>%

select(study_id, study, year, outcome,

mean_axpsa, sd_axpsa, n_axpsa,

mean_axspa, sd_axspa, n_axspa, n_total,

md, vi,

population_c, definition_axpsa, ctrl_as, ctrl_axspa,

def_mny, def_asas, def_other,

sex, age, dd, pa)

}

clean_binary <- function(df, outcome_name) {

df %>%

filter(!is.na(authors)) %>%

rename(study = authors, year = year,

event_axpsa = axpsa_event, total_axpsa = axpsa_total,

event_axspa = axspa_event, total_axspa = axspa_total,

population_c = population_c,

definition_axpsa = The.definition.of.axpsa,

sex = sex, age = age, dd = DD, pa = PA) %>%

mutate(outcome = outcome_name) %>%

mutate(across(where(is.character), ~na_if(., "NA"))) %>%

mutate(event_axpsa = as.numeric(event_axpsa), total_axpsa = as.numeric(total_axpsa),

event_axspa = as.numeric(event_axspa), total_axspa = as.numeric(total_axspa),

population_c = as.numeric(population_c),

definition_axpsa = as.numeric(definition_axpsa),

sex = as.numeric(sex), age = as.numeric(age),

dd = as.numeric(dd), pa = as.numeric(pa)) %>%

mutate(study_id = paste0(study, "_", year),

n_total = total_axpsa + total_axspa,

rr = ifelse(event_axpsa == 0 | event_axspa == 0, NA,

(event_axpsa / total_axpsa) / (event_axspa / total_axspa)),

log_rr = log(rr),

se_log_rr = sqrt((1/event_axpsa) - (1/total_axpsa) + (1/event_axspa) - (1/total_axspa)),

vi = se_log_rr^2,

ctrl_as = ifelse(population_c == 1, 1, 0),

ctrl_axspa = ifelse(population_c == 0, 1, 0),

def_mny = ifelse(definition_axpsa == 1, 1, 0),

def_asas = ifelse(definition_axpsa == 2, 1, 0),

def_other = ifelse(definition_axpsa == 3, 1, 0)) %>%

select(study_id, study, year, outcome,

event_axpsa, total_axpsa, event_axspa, total_axspa, n_total,

rr, log_rr, vi,

population_c, definition_axpsa, ctrl_as, ctrl_axspa,

def_mny, def_asas, def_other,

sex, age, dd, pa)

}

# Clean data

basdai <- clean_continuous(basdai_raw, "BASDAI")

asdas <- clean_continuous(asdas_crp_raw, "ASDAS_CRP")

basfi <- clean_continuous(basfi_raw, "BASFI")

crp <- clean_continuous(crp_raw, "CRP")

hla <- clean_binary(hla_raw, "HLA_B27")

continuous_data <- bind_rows(basdai, asdas, basfi, crp)

binary_data <- hla

# (Optional) Save cleaned data for external use

write.csv(continuous_data, "data_continuous.csv", row.names = FALSE, fileEncoding = "UTF-8")

write.csv(binary_data, "data_binary.csv", row.names = FALSE, fileEncoding = "UTF-8")

# =============================================================================

# Meta‑regression function (with Knapp‑Hartung correction)

# =============================================================================

run_meta_regression <- function(result, covariates) {

if (is.null(result)) return(NULL)

cat("\n", result$outcome, ":\n", sep = "")

reg_results <- list()

for (cov in covariates) {

if (!cov$var %in% names(result$data)) next

if (all(is.na(result$data[[cov$var]]))) next

formula_str <- paste0("~ ", cov$var)

tryCatch({

if (result$type == "continuous") {

res_mr <- rma.uni(yi = md, vi = vi, mods = as.formula(formula_str),

data = result$data, method = "REML", test = "knha")

cat(sprintf(" %s: beta = %.3f, p = %.4f\n",

cov$name, res_mr$b[2], res_mr$pval[2]))

} else {

res_mr <- rma.uni(yi = log_rr, vi = vi, mods = as.formula(formula_str),

data = result$data, method = "REML", test = "knha")

cat(sprintf(" %s: beta = %.3f, RR = %.3f, p = %.4f\n",

cov$name, res_mr$b[2], exp(res_mr$b[2]), res_mr$pval[2]))

}

reg_results[[cov$name]] <- res_mr

}, error = function(e) {

cat(sprintf(" %s: analysis failed (%s)\n", cov$name, e$message))

})

}

return(reg_results)

}

# Covariates list (as used in the original script)

covariates <- list(

list(name = "Control (AS)", var = "ctrl_as"),

list(name = "ax-PsA definition (mNY)", var = "def_mny"),

list(name = "ax-PsA definition (ASAS)", var = "def_asas"),

list(name = "Sample size", var = "n_total"),

list(name = "Sex", var = "sex"),

list(name = "Age", var = "age"),

list(name = "Disease duration", var = "dd"),

list(name = "Peripheral arthritis", var = "pa")

)

# Prepare data structure for regression (same as original run_meta_analysis but without primary meta‑analysis)

# We need results list with data and type for each outcome.

results <- list()

for (out in c("BASDAI", "ASDAS_CRP", "BASFI", "CRP")) {

dat <- continuous_data[continuous_data$outcome == out, ]

if (nrow(dat) > 0) {

results[[out]] <- list(data = dat, type = "continuous", outcome = out)

} else {

results[[out]] <- NULL

}

}

# HLA_B27 binary

dat_bin <- binary_data[binary_data$outcome == "HLA_B27", ]

if (nrow(dat_bin) > 0) {

results[["HLA_B27"]] <- list(data = dat_bin, type = "binary", outcome = "HLA_B27")

} else {

results[["HLA_B27"]] <- NULL

}

# Run meta‑regression for all outcomes

regression_results <- list()

for (outcome in names(results)) {

regression_results[[outcome]] <- run_meta_regression(results[[outcome]], covariates)

}

# =============================================================================

# Export meta‑regression results to CSV

# =============================================================================

regression_summary <- data.frame()

for (outcome in names(regression_results)) {

if (is.null(regression_results[[outcome]])) next

for (cov_name in names(regression_results[[outcome]])) {

res_mr <- regression_results[[outcome]][[cov_name]]

regression_summary <- rbind(regression_summary, data.frame(

Outcome = outcome,

Covariate = cov_name,

Beta = res_mr$b[2],

SE = res_mr$se[2],

CI_LB = res_mr$ci.lb[2],

CI_UB = res_mr$ci.ub[2],

Z = res_mr$zval[2],

P_Value = res_mr$pval[2],

stringsAsFactors = FALSE

))

}

}

write.csv(regression_summary, "meta_regression_results.csv", row.names = FALSE, fileEncoding = "UTF-8")

cat("\n========================================\n")

cat("Meta‑regression completed.\n")

cat("Results saved to:", file.path(work_dir, "meta_regression_results.csv"), "\n")

# =============================================================================

# Meta-regression visualization - multi-panel plots combined by outcome (suitable for appendix)

# Uses fixed path + Knapp-Hartung correction + patchwork layout

# =============================================================================

# Load packages (auto-install if missing)

pkgs <- c("metafor", "ggplot2", "data.table", "patchwork")

for (pkg in pkgs) {

if (!require(pkg, character.only = TRUE)) {

install.packages(pkg)

library(pkg, character.only = TRUE)

}

}

# =============================================================================

# 1. Set working directory (verify the path is correct)

# =============================================================================

work_dir <- "C:/Users/11638/Documents/meta_analysis"

if (!dir.exists(work_dir)) dir.create(work_dir, recursive = TRUE)

setwd(work_dir)

fig_dir <- file.path(work_dir, "figures", "meta_regression")

if (!dir.exists(fig_dir)) dir.create(fig_dir, recursive = TRUE)

cat("Working directory:", getwd(), "\n")

cat("Figure directory:", fig_dir, "\n")

# =============================================================================

# 2. Read data and check required columns

# =============================================================================

dt_cont <- as.data.frame(fread("data_continuous.csv", encoding = "UTF-8"))

dt_bin <- as.data.frame(fread("data_binary.csv", encoding = "UTF-8"))

required_cont <- c("outcome", "md", "vi", "sex", "age", "dd", "pa", "n_total")

required_bin <- c("outcome", "log_rr", "vi", "sex", "age", "dd", "pa", "n_total")

for (col in required_cont) {

if (!col %in% names(dt_cont)) stop("Continuous data missing column: ", col)

}

for (col in required_bin) {

if (!col %in% names(dt_bin)) stop("Binary data missing column: ", col)

}

for (col in required_cont[required_cont != "outcome"]) dt_cont[[col]] <- as.numeric(as.character(dt_cont[[col]]))

for (col in required_bin[required_bin != "outcome"]) dt_bin[[col]] <- as.numeric(as.character(dt_bin[[col]]))

cat("Continuous data rows:", nrow(dt_cont), "\n")

cat("Binary data rows:", nrow(dt_bin), "\n")

# =============================================================================

# 3. Function to check data validity for meta-regression

# =============================================================================

is_valid <- function(dat, covar, yi_name) {

if (!covar %in% names(dat)) return(FALSE)

complete_idx <- complete.cases(dat[, c(yi_name, "vi", covar)])

if (sum(complete_idx) < 3) {

cat(" Valid studies < 3, skipping\n")

return(FALSE)

}

if (length(unique(dat[[covar]][complete_idx])) < 2) {

cat(" Covariate has no variation, skipping\n")

return(FALSE)

}

if (any(dat$vi[complete_idx] <= 0)) {

cat(" Non-positive sampling variance detected, skipping\n")

return(FALSE)

}

return(TRUE)

}

# =============================================================================

# 4. Individual plotting functions (return ggplot objects, do not save)

# Using Knapp-Hartung correction (test="knha")

# =============================================================================

plot_bubble_cont <- function(outcome, cov_name, cov_col) {

dat <- dt_cont[dt_cont$outcome == outcome, ]

if (nrow(dat) == 0) return(NULL)

if (!is_valid(dat, cov_col, "md")) return(NULL)

res <- tryCatch(

rma(yi = md, vi = vi, mods = as.formula(paste0("~", cov_col)),

data = dat, method = "REML", test = "knha"),

error = function(e) { cat(" Model failed:", e$message, "\n"); NULL }

)

if (is.null(res)) return(NULL)

x_seq <- seq(min(dat[[cov_col]], na.rm=TRUE), max(dat[[cov_col]], na.rm=TRUE), length.out=100)

pred <- predict(res, newmods = x_seq)

plot_dat <- dat[complete.cases(dat[, c("md", "vi", cov_col)]), ]

plot_dat$weight <- 1 / sqrt(plot_dat$vi)

# Add significance marker

p_val <- res$pval[2]

sig_star <- ifelse(p_val < 0.001, "***", ifelse(p_val < 0.01, "**", ifelse(p_val < 0.05, "*", "")))

p <- ggplot(plot_dat, aes(x = .data[[cov_col]], y = md, size = weight)) +

geom_point(alpha = 0.6, color = "steelblue", shape = 21, fill = "lightblue") +

geom_ribbon(data = data.frame(x = x_seq, ymin = pred$ci.lb, ymax = pred$ci.ub),

aes(x = x, ymin = ymin, ymax = ymax),

fill = "gray80", alpha = 0.5, inherit.aes = FALSE) +

geom_line(data = data.frame(x = x_seq, y = pred$pred),

aes(x = x, y = y),

color = "darkred", linewidth = 1.2, inherit.aes = FALSE) +

scale_size(range = c(3,10), name = "Weight") +

labs(x = cov_name, y = "MD",

subtitle = sprintf("β = %.3f [%.3f, %.3f], p = %.4f %s",

res$b[2], res$ci.lb[2], res$ci.ub[2], p_val, sig_star)) +

theme_minimal(base_size = 10) +

theme(plot.subtitle = element_text(size = 9, color = "gray30"))

return(p)

}

plot_bubble_bin <- function(outcome, cov_name, cov_col) {

dat <- dt_bin[dt_bin$outcome == outcome, ]

if (nrow(dat) == 0) return(NULL)

if (!is_valid(dat, cov_col, "log_rr")) return(NULL)

res <- tryCatch(

rma(yi = log_rr, vi = vi, mods = as.formula(paste0("~", cov_col)),

data = dat, method = "REML", test = "knha"),

error = function(e) { cat(" Model failed:", e$message, "\n"); NULL }

)

if (is.null(res)) return(NULL)

x_seq <- seq(min(dat[[cov_col]], na.rm=TRUE), max(dat[[cov_col]], na.rm=TRUE), length.out=100)

pred <- predict(res, newmods = x_seq)

plot_dat <- dat[complete.cases(dat[, c("log_rr", "vi", cov_col)]), ]

plot_dat$weight <- 1 / sqrt(plot_dat$vi)

p_val <- res$pval[2]

sig_star <- ifelse(p_val < 0.001, "***", ifelse(p_val < 0.01, "**", ifelse(p_val < 0.05, "*", "")))

p <- ggplot(plot_dat, aes(x = .data[[cov_col]], y = log_rr, size = weight)) +

geom_point(alpha = 0.6, color = "darkred", shape = 21, fill = "pink") +

geom_ribbon(data = data.frame(x = x_seq, ymin = pred$ci.lb, ymax = pred$ci.ub),

aes(x = x, ymin = ymin, ymax = ymax),

fill = "gray80", alpha = 0.5, inherit.aes = FALSE) +

geom_line(data = data.frame(x = x_seq, y = pred$pred),

aes(x = x, y = y),

color = "navy", linewidth = 1.2, inherit.aes = FALSE) +

scale_size(range = c(3,10), name = "Weight") +

scale_y_continuous(breaks = c(-1,-0.5,0,0.5,1),

labels = c("0.37","0.61","1.00","1.65","2.72"),

sec.axis = dup_axis(name = "log(RR)")) +

labs(x = cov_name, y = "RR (log scale)",

subtitle = sprintf("β = %.3f [%.3f, %.3f], p = %.4f %s",

res$b[2], res$ci.lb[2], res$ci.ub[2], p_val, sig_star)) +

theme_minimal(base_size = 10) +

theme(plot.subtitle = element_text(size = 9, color = "gray30"))

return(p)

}

plot_pi_cont <- function(outcome, cov_name, cov_col) {

dat <- dt_cont[dt_cont$outcome == outcome, ]

if (!is_valid(dat, cov_col, "md")) return(NULL)

res <- tryCatch(

rma(yi = md, vi = vi, mods = as.formula(paste0("~", cov_col)),

data = dat, method = "REML", test = "knha"),

error = function(e) NULL

)

if (is.null(res)) return(NULL)

x_seq <- seq(min(dat[[cov_col]], na.rm=TRUE)*0.95, max(dat[[cov_col]], na.rm=TRUE)*1.05, length.out=200)

pred <- predict(res, newmods = x_seq)

pi_lb <- pred$pred - 1.96 * sqrt(pred$se^2 + res$tau2)

pi_ub <- pred$pred + 1.96 * sqrt(pred$se^2 + res$tau2)

plot_dat <- dat[complete.cases(dat[, c("md", "vi", cov_col)]), ]

p_val <- res$pval[2]

sig_star <- ifelse(p_val < 0.001, "***", ifelse(p_val < 0.01, "**", ifelse(p_val < 0.05, "*", "")))

p <- ggplot() +

geom_ribbon(data = data.frame(x = x_seq, ymin = pi_lb, ymax = pi_ub),

aes(x = x, ymin = ymin, ymax = ymax),

fill = "gray70", alpha = 0.4, inherit.aes = FALSE) +

geom_ribbon(data = data.frame(x = x_seq, ymin = pred$ci.lb, ymax = pred$ci.ub),

aes(x = x, ymin = ymin, ymax = ymax),

fill = "steelblue", alpha = 0.4, inherit.aes = FALSE) +

geom_line(data = data.frame(x = x_seq, y = pred$pred),

aes(x = x, y = y),

color = "darkblue", linewidth = 1.2, inherit.aes = FALSE) +

geom_point(data = plot_dat, aes(x = .data[[cov_col]], y = md),

alpha = 0.6, color = "darkred", size = 2, shape = 21, fill = "pink") +

labs(x = cov_name, y = "MD",

subtitle = sprintf("β = %.3f, τ² = %.4f, p = %.4f %s",

res$b[2], res$tau2, p_val, sig_star)) +

theme_minimal(base_size = 10) +

geom_hline(yintercept = 0, linetype = "dashed", color = "gray50") +

theme(plot.subtitle = element_text(size = 9, color = "gray30"))

return(p)

}

# =============================================================================

# 5. Generate combined plots (grouped by outcome)

# =============================================================================

# List of continuous outcomes

outcomes_cont <- c("BASDAI", "ASDAS_CRP", "BASFI", "CRP")

# Covariates list (for bubble plots)

covs_all <- list(

list(name = "Sex (Male %)", col = "sex"),

list(name = "Age (years)", col = "age"),

list(name = "Disease duration (DD)", col = "dd"),

list(name = "Peripheral arthritis (PA %)", col = "pa"),

list(name = "Sample size (N)", col = "n_total")

)

# Covariates for prediction interval plots (sex, disease duration, peripheral arthritis)

covs_pi <- covs_all[1:3]

# Generate combined bubble plots for continuous outcomes

for (out in outcomes_cont) {

cat("\nProcessing outcome:", out, "(bubble plot)\n")

plot_list <- list()

for (cv in covs_all) {

p <- plot_bubble_cont(out, cv$name, cv$col)

if (!is.null(p)) plot_list[[length(plot_list)+1]] <- p

}

if (length(plot_list) == 0) {

cat(" No subplots generated, skipping\n")

next

}

# patchwork layout: 2 columns, rows = ceiling(length(plot_list)/2)

combined <- wrap_plots(plot_list, ncol = 2) +

plot_annotation(title = paste(out, "- Meta-regression bubble plots (continuous covariates)"),

theme = theme(plot.title = element_text(size = 14, face = "bold")))

fname <- file.path(fig_dir, paste0("bubble_", out, "_all.pdf"))

ggsave(fname, combined, width = 12, height = 4 * ceiling(length(plot_list)/2), dpi = 150)

cat(" ✓ Saved:", basename(fname), "\n")

}

# Generate combined prediction interval plots for continuous outcomes (only sex, DD, PA)

for (out in outcomes_cont) {

cat("\nProcessing outcome:", out, "(prediction interval plot)\n")

plot_list <- list()

for (cv in covs_pi) {

p <- plot_pi_cont(out, cv$name, cv$col)

if (!is.null(p)) plot_list[[length(plot_list)+1]] <- p

}

if (length(plot_list) == 0) {

cat(" No subplots generated, skipping\n")

next

}

combined <- wrap_plots(plot_list, ncol = 2) +

plot_annotation(title = paste(out, "- Meta-regression prediction interval plots"),

theme = theme(plot.title = element_text(size = 14, face = "bold")))

fname <- file.path(fig_dir, paste0("pi_", out, "_all.pdf"))

ggsave(fname, combined, width = 12, height = 4 * ceiling(length(plot_list)/2), dpi = 150)

cat(" ✓ Saved:", basename(fname), "\n")

}

# =============================================================================

# 6. HLA_B27 binary outcome

# =============================================================================

cat("\nProcessing HLA_B27 (bubble plot)\n")

plot_list_bin <- list()

for (cv in covs_all) {

p <- plot_bubble_bin("HLA_B27", cv$name, cv$col)

if (!is.null(p)) plot_list_bin[[length(plot_list_bin)+1]] <- p

}

if (length(plot_list_bin) > 0) {

combined_bin <- wrap_plots(plot_list_bin, ncol = 2) +

plot_annotation(title = "HLA-B27 - Meta-regression bubble plots (continuous covariates)",

theme = theme(plot.title = element_text(size = 14, face = "bold")))

fname <- file.path(fig_dir, "bubble_HLA_B27_all.pdf")

ggsave(fname, combined_bin, width = 12, height = 4 * ceiling(length(plot_list_bin)/2), dpi = 150)

cat(" ✓ Saved:", basename(fname), "\n")

}

cat("\nProcessing HLA_B27 (prediction interval plot)\n")

plot_list_pi_bin <- list()

for (cv in covs_pi) {

# Note: plot_pi_cont cannot be used for binary outcome; define plot_pi_bin locally

plot_pi_bin <- function(outcome, cov_name, cov_col) {

dat <- dt_bin[dt_bin$outcome == outcome, ]

if (!is_valid(dat, cov_col, "log_rr")) return(NULL)

res <- tryCatch(

rma(yi = log_rr, vi = vi, mods = as.formula(paste0("~", cov_col)),

data = dat, method = "REML", test = "knha"),

error = function(e) NULL

)

if (is.null(res)) return(NULL)

x_seq <- seq(min(dat[[cov_col]], na.rm=TRUE)*0.95, max(dat[[cov_col]], na.rm=TRUE)*1.05, length.out=200)

pred <- predict(res, newmods = x_seq)

pi_lb <- pred$pred - 1.96 * sqrt(pred$se^2 + res$tau2)

pi_ub <- pred$pred + 1.96 * sqrt(pred$se^2 + res$tau2)

plot_dat <- dat[complete.cases(dat[, c("log_rr", "vi", cov_col)]), ]

p_val <- res$pval[2]

sig_star <- ifelse(p_val < 0.001, "***", ifelse(p_val < 0.01, "**", ifelse(p_val < 0.05, "*", "")))

p <- ggplot() +

geom_ribbon(data = data.frame(x = x_seq, ymin = pi_lb, ymax = pi_ub),

aes(x = x, ymin = ymin, ymax = ymax),

fill = "gray70", alpha = 0.4, inherit.aes = FALSE) +

geom_ribbon(data = data.frame(x = x_seq, ymin = pred$ci.lb, ymax = pred$ci.ub),

aes(x = x, ymin = ymin, ymax = ymax),

fill = "steelblue", alpha = 0.4, inherit.aes = FALSE) +

geom_line(data = data.frame(x = x_seq, y = pred$pred),

aes(x = x, y = y),

color = "darkblue", linewidth = 1.2, inherit.aes = FALSE) +

geom_point(data = plot_dat, aes(x = .data[[cov_col]], y = log_rr),

alpha = 0.6, color = "darkred", size = 2, shape = 21, fill = "pink") +

labs(x = cov_name, y = "log(RR)",

subtitle = sprintf("β = %.3f, τ² = %.4f, p = %.4f %s",

res$b[2], res$tau2, p_val, sig_star)) +

theme_minimal(base_size = 10) +

geom_hline(yintercept = 0, linetype = "dashed", color = "gray50") +

theme(plot.subtitle = element_text(size = 9, color = "gray30"))

return(p)

}

p <- plot_pi_bin("HLA_B27", cv$name, cv$col)

if (!is.null(p)) plot_list_pi_bin[[length(plot_list_pi_bin)+1]] <- p

}

if (length(plot_list_pi_bin) > 0) {

combined_pi_bin <- wrap_plots(plot_list_pi_bin, ncol = 2) +

plot_annotation(title = "HLA-B27 - Meta-regression prediction interval plots",

theme = theme(plot.title = element_text(size = 14, face = "bold")))

fname <- file.path(fig_dir, "pi_HLA_B27_all.pdf")

ggsave(fname, combined_pi_bin, width = 12, height = 4 * ceiling(length(plot_list_pi_bin)/2), dpi = 150)

cat(" ✓ Saved:", basename(fname), "\n")

}

cat("\nDone! All combined figures saved in:", fig_dir, "\n")
